# Protection of Double-Stranded RNA *via* Complexation with Double Hydrophilic Block Copolymers: Influence of Neutral Block Length in Biologically Relevant Environments

**DOI:** 10.1021/acs.biomac.2c00136

**Published:** 2022-05-12

**Authors:** Charlotte E. Pugsley, R. Elwyn Isaac, Nicholas. J. Warren, Juliette S. Behra, Kaat Cappelle, Rosa Dominguez-Espinosa, Olivier. J. Cayre

**Affiliations:** † School of Chemical and Process Engineering, University of Leeds, Leeds LS2 9JT, United Kingdom; ‡ School of Biology, Faculty of Biological Sciences, University of Leeds, Leeds LS2 9JT, United Kingdom; § Syngenta Ghent Innovation Center, Technologiepark 30, B-9052 Gent-Zwijnaarde, Belgium; ∥ Syngenta Jealott’s Hill International Research Centre, Bracknell, Berkshire RG42 6EY, England

## Abstract

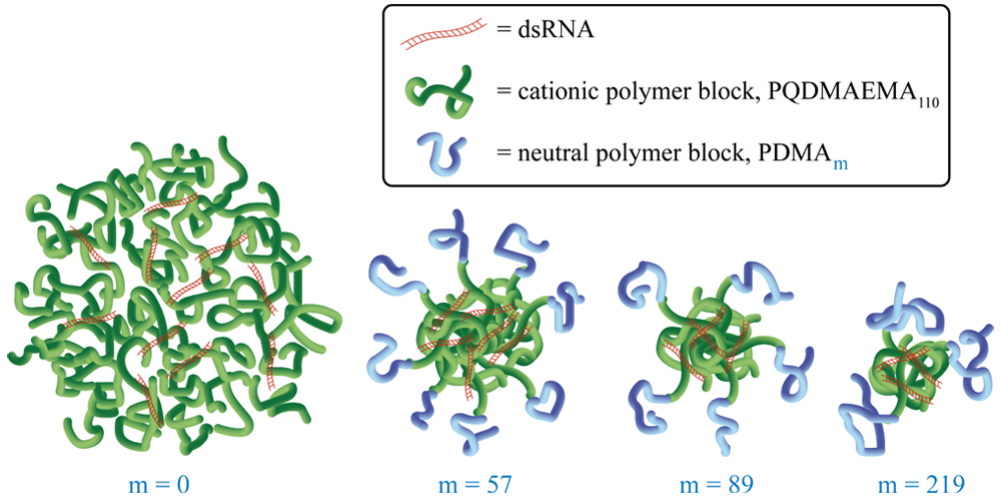

Interaction between the anionic phosphodiester backbone of DNA/RNA and polycations can be exploited as a means of delivering genetic material for therapeutic and agrochemical applications. In this work, quaternized poly(2-(dimethylamino)ethyl methacrylate)-*block*-poly(*N*,*N*-dimethylacrylamide) (PQDMAEMA-*b*-PDMA_m_) double hydrophilic block copolymers (DHBCs) were synthesized *via* reversible addition–fragmentation chain-transfer (RAFT) polymerization as nonviral delivery vehicles for double-stranded RNA. The assembly of DHBCs and dsRNA forms distinct polyplexes that were thoroughly characterized to establish a relationship between the length of the uncharged poly(*N,N*-dimethylacrylamide) (PDMA) block and the polyplex size, complexation efficiency, and colloidal stability. Dynamic light scattering reveals the formation of smaller polyplexes with increasing PDMA lengths, while gel electrophoresis confirms that these polyplexes require higher N/P ratio for full complexation. DHBC polyplexes exhibit enhanced stability in low ionic strength environments in comparison to homopolymer-based polyplexes. *In vitro* enzymatic degradation assays demonstrate that both homopolymer and DHBC polymers efficiently protect dsRNA from degradation by RNase A enzyme.

## Introduction

Cationic polymers can electrostatically interact with the anionic phosphodiester backbone of nucleic acids such as DNA or RNA to form “polyplexes”. This interaction is entropically favorable due to the release of small counterions upon complexation and has thus been widely exploited for the delivery of genetic material.^1,2^ Transportation of exogenous DNA or RNA into cells has long been of interest in the therapeutic field for application in gene therapy, where viral delivery vehicles for DNA/RNA were initially adopted due to their high transfection rates.^3−5^ However, issues of immunogenicity and tumor development have provided motivation for research to focus on nonviral delivery vehicles, such as cationic polymers.^6−10^ Another area of practical interest for nucleic acid delivery is the agrochemical industry and, in particular, the use of double-stranded RNA (dsRNA) as a species-specific bioinsecticide by triggering the naturally occurring RNA interference (RNAi) mechanism in the target pests.^11−14^ Polymeric delivery vehicles are of particular value here. Indeed, despite systemic RNAi being demonstrated in a number of pest insect species, the administered dsRNA degrades prior to inducing RNAi effects in more recalcitrant species, highlighting the need to protect dsRNA during delivery to crops and insects.^15−17^

Typically, polycations such as polyethylenimine (PEI) or poly(2-(dimethylamino)ethyl methacrylate) (PDMAEMA) have been employed for gene delivery.^18−23^ PEI and PDMAEMA contain amine groups capable of protonation at physiological pH and have, as a result, favorable electrostatic interaction with DNA/RNA phosphate groups driving efficient complexation. However, cationic homopolymers can exhibit high levels of cytotoxicity, and the polyplexes they form with DNA/RNA can be unstable, with the likeliness of electroneutralization upon complexation with DNA/RNA leading to increased aggregation.^6,20−22,24−26^ Thus, tailored polymer architectures including branched,^16,27,28^ dendritic,^29^ or block copolymers^30−33^ have recently been evaluated for improving the stabilization of polyplexes for the protection and targeted release of genetic material.

In this work, we focus our attention on double hydrophilic block copolymers (DHBCs) for the stabilization and protection of dsRNA upon complexation. We have synthesized novel DHBCs *via* aqueous RAFT polymerization of quaternized poly(2-(dimethylamino)ethyl methacrylate) (PQDMAEMA) and poly(*N*,*N*-dimethylacrylamide) (PDMA). In designing these diblock copolymer structures, we hypothesize that condensation of the dsRNA by PQDMAEMA will form the interpolyelectrolyte core of the polyplex, with PDMA forming a corona that provides steric stabilization preventing aggregation between the formed polyplexes.^34^ Hydrophilicity of both cationic and neutral blocks enhances the biocompatibility and dispersibility of polyplexes.^2,35^ Indeed, prior experimental studies, as well as coarse-grained simulation, have indicated that hydrophilic charge-neutral blocks incorporated alongside the cationic element can significantly impact polyplex morphology, stability, and transfection efficiency.^35−39^ Typically, a poly(ethylene glycol) (PEG) chain has been used as a polyplex stabilizing block.^26,40−42^ Some studies, however, suggest that PEG reduces the cellular uptake of siRNA/DNA.^43−45^ Therefore, PDMA was chosen as an alternative polymer block due to its uncharged, hydrophilic, and biocompatible nature.^46^

Aqueous RAFT polymerization enables facile control over polymer length, allowing the tailored variation of the PDMA block length. This allowed for a systematic comparison of the physicochemical properties of different polymer architectures, including their impact on the morphology and stability of the resulting polyplexes with dsRNA. In both therapeutic and agrochemical applications, the influence of environmental conditions such as electrolyte concentration, driving competitive adsorption/desorption of counterions, and the presence of nuclease enzymes should be taken into account to create an effective formulation of DNA/RNA polyplexes. In this work, we specifically probe the impact of changes in polyplex size, stability, and efficiency in protecting the dsRNA as a function of the diblock copolymer characteristics using dynamic light scattering (DLS), fluorescence spectroscopy, electrophoretic mobility assays, and agarose gel electrophoresis.

## Experimental Section

### Materials

[2-(Methacryloyloxy)ethyl] trimethylammonium chloride solution (QDMAEMA, 80 wt % in H_2_O), *N*,*N*-dimethylacrylamide (DMA, 99%), sodium chloride (NaCl, 99.5%), D_2_O (99.9%), and hydrochloric acid (HCl, 12 M) were purchased from Sigma-Aldrich. 4-((((2-Carboxyethyl)thio)carbonothioyl)thio)-4-cyano-pentanoic acid (CCCP, 95%) was purchased from Boron Molecular. 4,4′-Azobis(4-cyanovaleric acid) (ACVA, 97%) was purchased from Acros Organics. V-ATPase 222 bp dsRNA was synthesized by Genolution AgroRNA (4.68 μg μL^–1^), sequence-specific to the pest insect, *Drosophila suzukii*. Ethidium bromide (EB, 10 mg mL^–1^) and regenerated cellulose dialysis, with a membrane molecular weight cutoff (MWCO) < 3500 g mol^–1^, were purchased from Fisher Scientific. DNA ladder (100 bp, 500 μg mL^–1^) and RNase A (20 mg mL^–1^) were purchased from New England Biolabs. Blue/orange loading dye (6×) was purchased from Promega. Ultrapure Milli-Q water (resistivity of minimum 18.2 MΩ·cm) was used for solution preparation and dialysis, and nuclease-free water was used for biological assays.

### Synthesis of PQDMAEMA Macromolecular-Chain-Transfer Agent (Macro-CTA)

**Figure 1:**
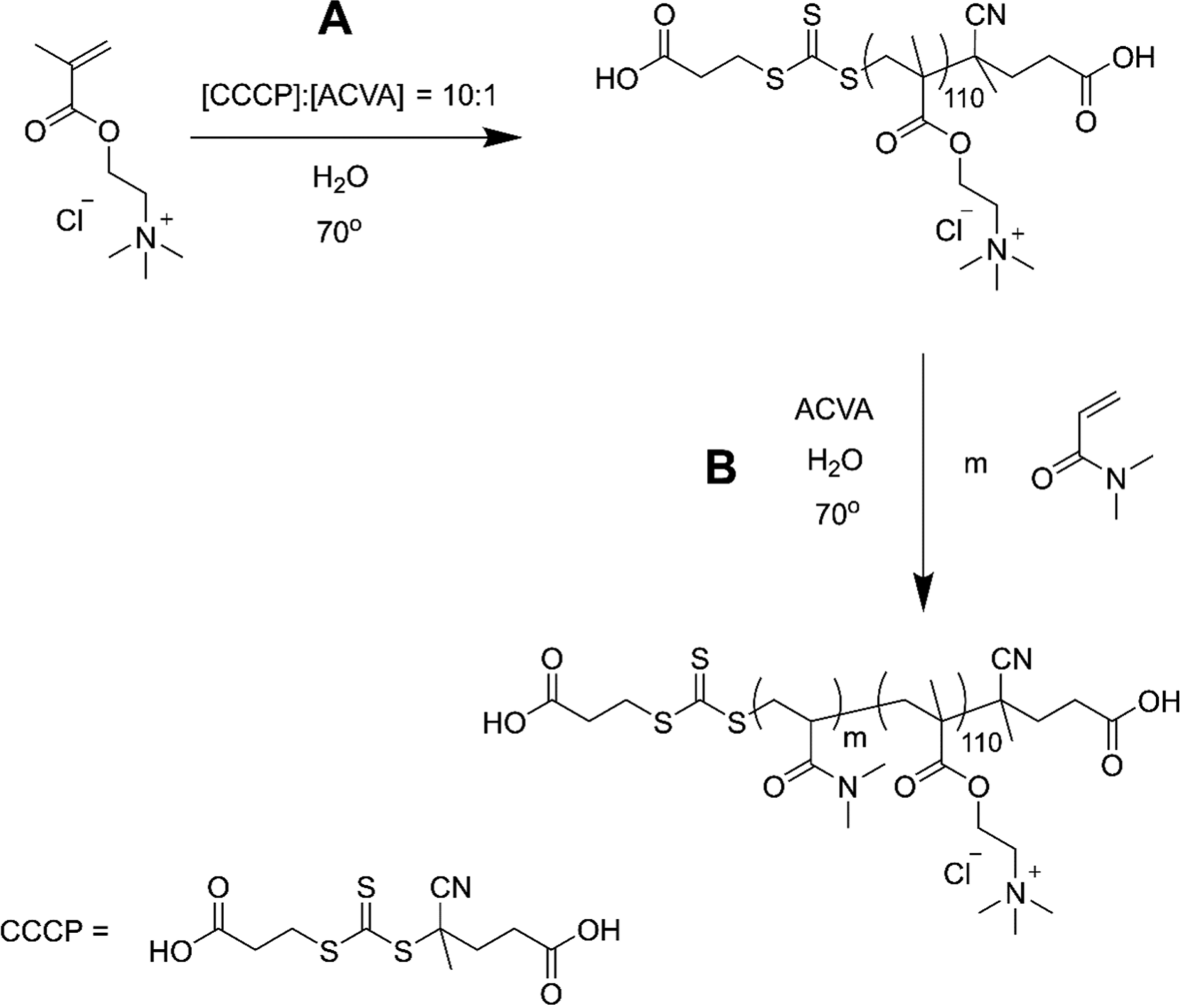
Reaction scheme of RAFT polymerization of the (A) PQDMAEMA macro-CTA (Q_110_) and subsequently the (B) PQDMAEMA_110_-*b*-PDMA_m_ (Q_110_-*b*-D_m_) double hydrophilic block copolymers.

The PQDMAEMA macro-CTA was synthesized by RAFT polymerization, as shown in the scheme in Figure 1A. QDMAEMA (100 g, 80 wt % in H_2_O, 385 mmol), CCCP (0.94 g, 3.1 mmol), and ACVA (0.086 g, 0.31 mmol) were dissolved in Milli-Q water at a ratio of [QDMAEMA]:[CCCP]:[ACVA] = 126:1:0.1 and 50 wt % in solution, pH = 4.3. The solution was degassed with N_2_ for 45 min and then stirred at 70 °C for 1.5 h. The reaction was quenched by exposure to air. PQDMAEMA macro-CTA was stored at −20 °C to prevent degradation of RAFT chain-end groups, prior to purification by dialysis against Milli-Q water (MWCO < 3500 g mol^–1^) and lyophilization. The degree of polymerization (DP), 110, and conversion, 88%, were confirmed by ^1^H NMR spectroscopy (400 MHz) through comparison of a peak from the pendant amine group (b) to a peak from the RAFT-end group (d), as demonstrated by Figure S1 in the Supporting Information (SI).

### Synthesis of PQDMAEMA-*b*-PDMA

Double hydrophilic block copolymers were synthesized through chain extension of the previously synthesized PQDMAEMA_110_ macro-CTA, as shown in the scheme in Figure 1B. PQDMAEMA_110_ macro-CTA (10 g, 0.38 mmol), ACVA (0.01 g, 0.038 mmol), and DMA (amount varied to control the degree of polymerization) were dissolved in Milli-Q water at a ratio of [macro-CTA]:[ACVA] = 1:0.1 and 50 wt % in solution, pH = 6.6. The concentration of the DMA monomer was varied to control the length of the DMA block. The solutions were degassed with N_2_ for 45 min and then stirred at 70 °C for 4 h. The reactions were quenched by exposure to air. The solutions were then purified by dialysis against Milli-Q water to remove the unreacted monomer (MWCO < 3,500 g mol^–1^) and lyophilized to yield the PQDMAEMA-*b*-PDMA block copolymers as a (pale yellow) powder. The DP (57, 89, and 219) and conversion (86%, 71%, and 91%, respectively) were confirmed by ^1^H NMR spectroscopy (400 MHz) through comparison of a characteristic peak from the PDMA block (f) with a peak from the pendant amine group (b) (see Figure S1 in the SI).

### Polymer Characterization

#### Gel Permeation Chromatography (GPC)

Molecular weight (MW) and molar mass dispersity (*Đ*) of the polymers were ascertained by aqueous GPC, using an Agilent 1260 Infinity 2 instrument equipped with a refractive index detector. Separation was achieved using two PL aquagel-OH Mixed-H columns and an 8 μm guard column (Agilent Technologies). The eluent comprised 0.8 M NaNO_3_, 0.01 M NaH_2_PO_4_, and 0.05 wt % NaN_3_ in Milli-Q water, adjusted to pH 3 using 37% (w/w) HCl. It was eluted at a rate of 1.0 mL min^–1^. Samples were diluted to 0.5 mg mL^–1^ in the eluent and filtered through a 0.2 μm syringe filter (Sartorius Minisart RC hydrophilic) prior to analysis. MW was calibrated against poly(ethylene glycol)/poly(ethylene oxide) (PEG/PEO) standards with molecular weights varying from 106 to 1,500,000 g mol^–1^ (EasiVial PEG/PEO calibration kit, PL2080-0201, Agilent Technologies).^47^

#### ^1^H NMR Spectroscopy

Composition analysis was conducted following the measurement of samples in D_2_O (5 mg mL^–1^) using a Bruker 400 MHz instrument after purification and lyophilization.

### Preparation of Q_110_/dsRNA and DHBC/dsRNA Polyplexes

DHBC and Q_110_ stock solutions were prepared by dissolving a known mass of polymer in the appropriate volume of Milli-Q water. Solutions were stirred at ∼800 rpm for 5 min to ensure complete dissolution. DsRNA solutions were prepared through dilution of the 4.68 g L^–1^ stock solution with DNase and RNase-free water. Q_110_ or DHBC/dsRNA polyplexes were formulated by directly mixing specific volumes of the polymer solution and the dsRNA solution to achieve a desired N/P ratio. The N/P ratio expresses the ratio between the number of ammonium groups present in the PQDMAEMA homopolymer or the DHBCs (as determined through ^1^H NMR analysis) and the number of phosphate groups present in the dsRNA (222 bp, providing 444 phosphate groups per molecule). Polycation (Q_110_ or DHBC) was added to dsRNA solution, which was subsequently mixed thoroughly, before incubating at room temperature (RT) for at least 1.5 h to allow equilibration. The pH of the formulations was found to be 7.4.

### Dynamic Light Scattering (DLS)

To prevent dust contamination upon sample preparation, all glass vials, lids, and stirrer bars were washed 3× with filtered ultrapure Milli-Q water (filtered through two 0.2 μm pore-size nylon membrane nonsterile Fisherbrand filters mounted in series) and filtered isopropanol (IPA) (filtered through two 0.2 μm pore-size poly(tetrafluoroethylene) (PTFE) membrane nonsterile Fisherbrand filters mounted in series) before drying at ∼50 °C in a dust-free environment. Prior to measurements, samples were filtered to remove dust contamination through a 0.8 μm pore-size surfactant-free cellulose acetate membrane (Sartorius) into the prewashed (as described above) glass light scattering (LS) tubes (rimless Pyrex culture tubes 75 mm × 10 mm). Polymer and dsRNA solutions were prepared through dilution of a mother solution 48 h before measurement. Polyplexes were formulated at a low concentration (0.1 g L^–1^) approx. 24 h before measurement to allow for equilibration. DLS experiments were performed with a three-dimensional (3D) LS spectrometer (LS instruments, Switzerland) using the “two-dimensional (2D) mode”. The spectrometer is fitted with a diode-pumped solid-state (DPSS) laser operating at 660 nm with a maximum power of 105 mW (Cobolt FlamencoTM, Cobalt). Laser attenuation was automated, and two avalanche photodiode detectors were used; the light was vertically polarized. All experiments were performed at a temperature of 25 ± 0.5 °C controlled by a water bath. A pseudo cross-correlation mode was used. The angle of measurement was altered from 30 to 130°, and the associated scattering vector was calculated using eq 1. Fitting of the data was performed using the Levenberg–Marquardt algorithm, with details described in the Polyplex Formation and Size Analysissection
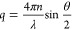
1where *q* is the scattering vector, *n* is the refractive index of the solvent, λ is the wavelength, and θ is the angle of detection.

For experiments where the salt concentration was varied, a ζ potential analyzer (Zetasizer Nano-ZS, Malvern) was used. A backscatter (173°) detection angle was used, with measurements performed in quintuplicate. Data fitting was performed as described in the Polyplex Formation and Size Analysis section.

### Electrophoretic Mobility

Electrophoretic mobility was measured at 25 ± 0.5 °C using the phase analysis light scattering technique. Measurements were carried out using a standard folded capillary cell (DTS1070, Malvern) with a ζ potential analyzer (Zetasizer Nano-ZS, Malvern). Samples were kept at RT, and their pH was measured at 7.5 ± 0.5. Data were collected in triplicate with the average taken over three runs. ζ potential, when used, was calculated by the instrument as determined by the Henry equation using the Smoluchowski approximation. Aqueous suspensions were prepared at a concentration of 0.1 g L^–1^ 24 h before measurement.

### Agarose Gel Electrophoresis

Aliquots of Q_110_ or DHBC were added to 1 μg dsRNA in quantities to vary the N/P ratio, with solutions left to incubate at RT for 1.5 h to allow for complexation. Two microliters of 6× blue/orange loading dye were added to each sample, and each solution (∼17 μL) was loaded onto a 2% (w/w) agarose gel containing 3.5 μL of EB, prepared with 1× TAE (tris base, acetic acid, and EDTA) buffer. Assays were run for 25 min at 90 V. A 1 μL aliquot of 100 bp DNA ladder, alongside 1 μL of 6× purple non-SDS dye and 4 μL of nuclease-free water, was run for comparison. The gel was imaged under a UV transilluminator at 365 nm. After RNase A (0.5 μL, 5 mg mL^–1^) was added to the polyplex solutions, the samples were incubated at 37 °C for 30 min prior to analysis.

### Fluorescence Spectroscopy

Ethidium bromide (EB) was used as a nucleic acid-intercalating fluorophore. EB solution was stored in an opaque container at 4 °C prior to use. Fluorescence intensity was detected for the EB exclusion assay and RNase A degradation profiles using an Omega FLUOstar (BMG LABTECH GmbH) multimode microplate reader, with λ_ex_ set at 320 nm and λ_em_ set at 594 nm. Samples were measured in a Corning Costar 96-well opaque microplate. Gain was set at 1600–1900.

For equilibrated static samples, endpoint measurements were taken with 10 flashes per well. The volume of each well was made up to 200 μL with nuclease-free water. For all samples, 8 μL (0.468 g L^–1^) of dsRNA were added to each well alongside 2.9 μL of EB (0.4 mg mL^–1^) that provided sufficient fluorescence intensity with the Omega FLUOstar (BMG LABTECH GmbH) at the ratio [EB]:[P] = 0.12 (molar concentration of EB in relation to the molar concentration of dsRNA phosphate groups, approximately one molecule of intercalated EB per four pairs of dsRNA bases). The dsRNA–EB solutions were left to incubate for at least 10 min prior to analysis for full intercalation of EB. In the EB exclusion assay, an equilibration time was incorporated after each polymer addition prior to endpoint measurements.

Fluorescence intensity (*F*_I_) was normalized using eq 2 with respect to the fluorescence intensity of dsRNA–EB alone (*F*_0_), subtracting the weak fluorescence intensity of EB in water (*F*_EB_)
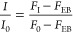
2For time-resolved studies, two flashes were used for each sample in the 6–8 s measurement cycle. Aliquots of Q_110_ or DHBC solution (1 g L^–1^) were added to dsRNA (8 μL, 1 g L^–1^) to achieve a specific N/P ratio 1.5 h prior to measurement. The volume of each well was made up to 200 μL with nuclease-free water. One microliter of EB (0.4 mg mL^–1^) and 1 μL of RNase A (5 mg mL^–1^) were added immediately prior to analysis if required. The incubator was set to 37.0 °C. The data were normalized with respect to fluorescence intensity at *t* = 0.

For fluorimetric NaCl titration assays, fluorescence intensity was detected using a FluoroMax (Horiba Scientific) spectrofluorometer, with λ_ex_ set at 320 nm and λ_em_ measured over a 335–800 nm window. Polyplex samples were prepared before analysis, with aliquots of Q_110_ or DHBC added to dsRNA (120 μL, 1 g L^–1^) to achieve an N/P ratio = 5. After incubation at RT for 1.5 h, nuclease-free water was added to *ca*. 3 mL and EB (91.5 μL) to provide [EB]:[P] = 0.25 (approximately one molecule of intercalated EB per two pairs of dsRNA bases). An equilibration time of 5 min was incorporated after each NaCl addition and prior to measurement.

## Results and Discussion

### Homopolymer and DHBC Aqueous RAFT Polymerization

**Figure 2:**
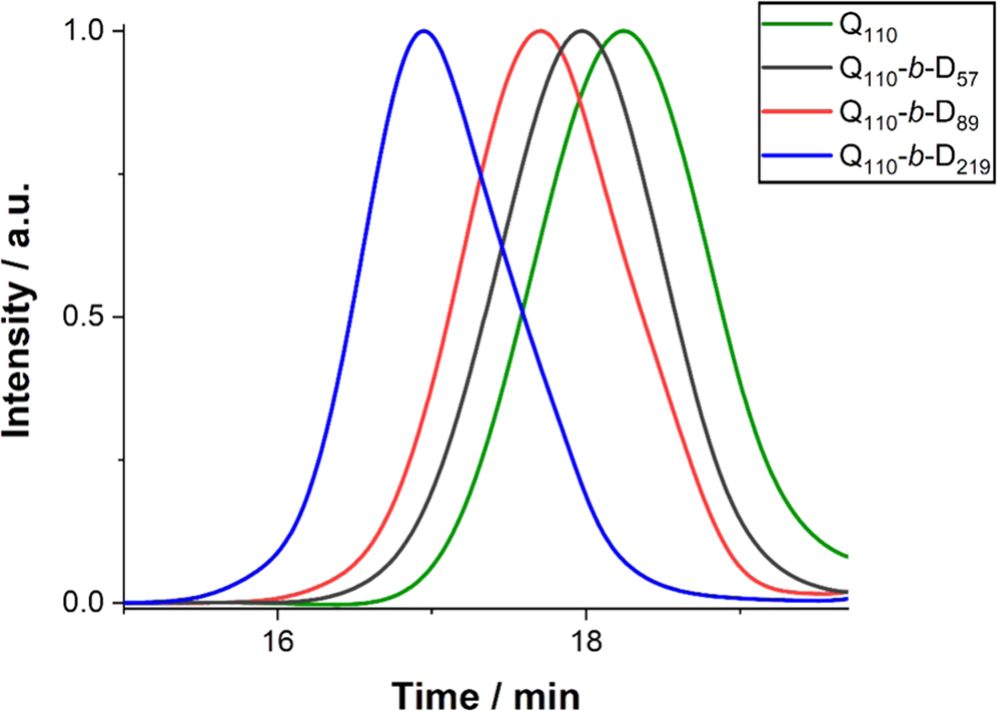
GPC chromatogram obtained for the PQDMAEMA macro-CTA and the series of three DHBCs with varying PDMA DPs. The *y*-axis represents the arbitrary-normalized signal from the RI detector.

A series of three double hydrophilic block copolymers (DHBCs) were synthesized by RAFT polymerization in aqueous media, as described in Figure 1. A cationic block of quaternized poly(2-(dimethylamino)ethyl methacrylate) (PQDMAEMA_110_, *M*_n_ = 23,100 g mol^–1^) was first synthesized by polymerizing QDMAEMA in the presence of 4-((((2-carboxyethyl)thio)carbonothioyl)thio)-4-cyano-pentanoic acid (CCCP). The dsRNA used in our work is significantly longer than the siRNA often used in studies concerned with therapeutic applications.^30,31,39,44^ On that basis, we decided to explore higher degrees of polymerization (DP) for the charged block than that used in the literature for siRNA. Unpublished data from our research group subsequently indicated that a PQDMAEMA DP of 110 was a sufficient length for complexation with the 222 bp dsRNA. The resulting macro-CTA was chain-extended with *N,N*-dimethylacrylamide (DMA) in water, where the ratio of DMA monomer to PQDMAEMA macro-CTA was varied to tailor the length of the neutral PDMA block as approximately half, equal, and double the length of the cationic block (Q_110_-*b*-D_57_, Q_110_-*b*-D_89_, and Q_110_-*b*-D_219_ with *M*_n_ = 28,800; 31,900; and 44,800 g mol^–1^, respectively). By polymerizing PQDMAEMA as a macro-CTA, a constant cationic block length was kept between homopolymer and diblock copolymers, maintaining the same size ratio of dsRNA to cationic charge in experiments. The influence of the neutral block size for the three DHBCs was thus investigated in terms of the polymer physicochemical properties, complexation efficiency, and the stability of polyplexes formed with dsRNA. The cationic homopolymer PQDMAEMA was purified and lyophilized separately. The homopolymer and DHBC compositions and molecular weights were determined by ^1^H NMR spectroscopy (400 MHz; see Figure S1 in the SI), as detailed in Table 1. Molecular composition was calculated through comparison of the relative intensity of an integrated PDMA peak (∼2.60 ppm) to the intensity of an integrated PQDMAEMA peak (∼3.80 ppm). Since our polymers are chemically different as compared to the polymer standards (*i.e*., PEG/PEO) used to calibrate the aqueous GPC system, their interactions with the column are expected to be different and the molecular weight values obtained with this technique can only be considered as relative values.^48^ Thus, to calculate N/P ratios in polyplex formulations, molecular weights derived from ^1^H NMR spectra were used. Aqueous GPC (Figure 2) showed the negligible presence of residual PQDMAEMA macro-CTA after chain extension, indicating good blocking efficiencies. Molar mass dispersity (*Đ*) of the DHBCs ranged from 1.23 to 1.39 (Table 1). These values are relatively high with respect to controlled RAFT polymerizations, where typical *Đ* values are 1.1–1.3^49^ but are suitable for our intended application.

**Table 1:** Properties of initial homopolymer (or macro-CTA) and the three DHBCs synthesized in this work

	Data determined by ^1^H NMR spectroscopy (400 MHz)			Data determined by aqueous GPC	
Polymer code	Monomer conversion (%)	PDMA proportion in DHBC (mol %)	*M*_n_ by NMR^a^ (g mol^–1^)	*M*_n_ by GPC (g mol^–1^)	*Đ*
Q_110_ (macro-CTA)	88	0	23,100	8,900	1.48
Q_110_-*b*-D_57_	86	34	28,800	12,600	1.39
Q_110_-*b*-D_89_	71	45	31,900	16,400	1.33
Q_110_-*b*-D_219_	91	67	44,800	31,600	1.23

a MW calculated using the following equation
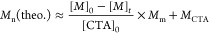
where [*M*]_0_ is the initial monomer concentration, [*M*]_*t*_ is the monomer concentration at time *t*, [CTA]_0_ is the initial CTA concentration, *M*_m_ is the molar mass of the monomer, and *M*_CTA_ is the molar mass of CTA.

### Polyplex Formation and Size Analysis

**Figure 3:**
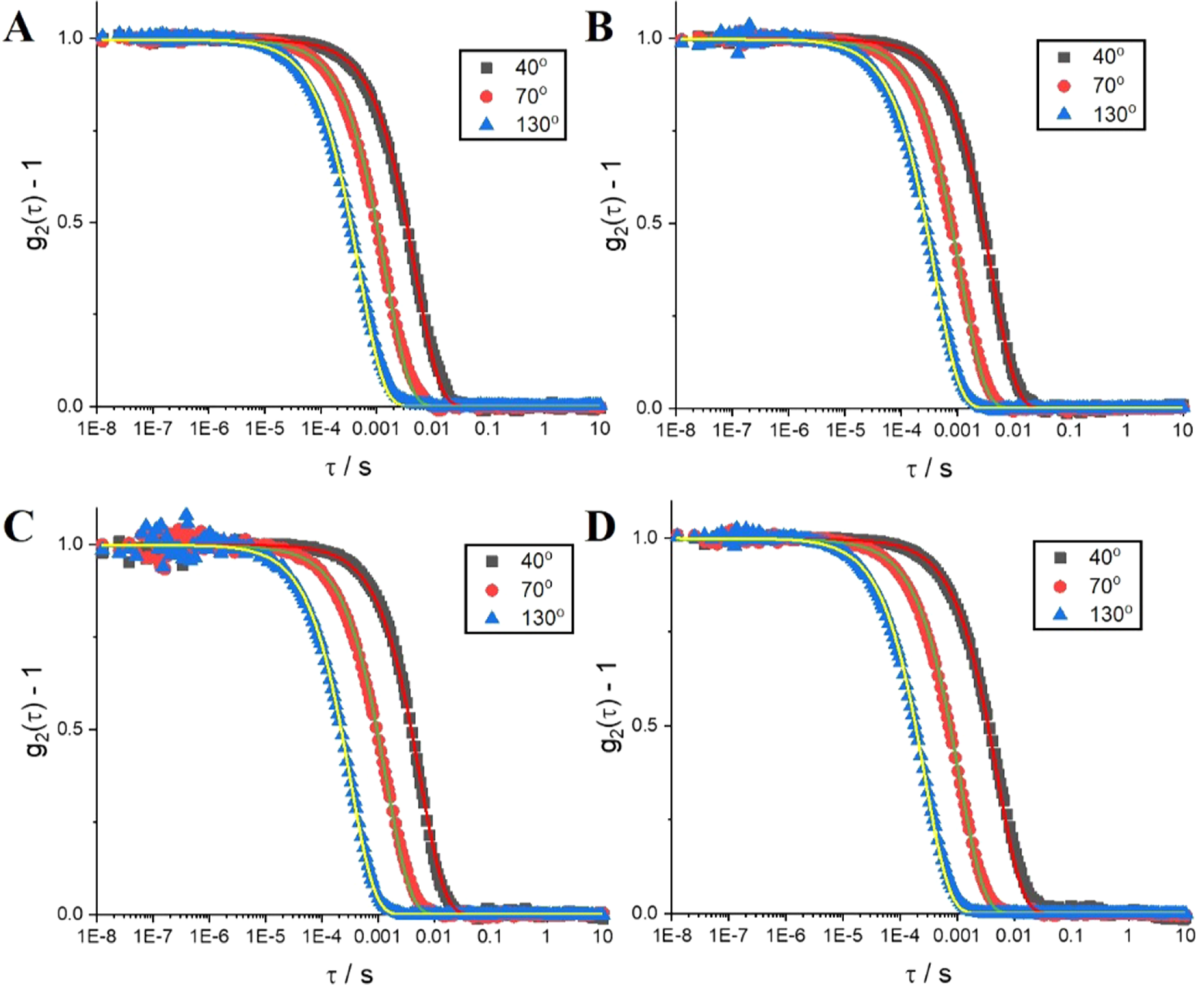
Normalized IAC data with single exponential fits obtained with eq 3 (continuous lines) for 40, 70, and 130° scattering angles of polyplexes with polymers of increasing PDMA chain lengths: (A) Q_110_, (B) Q_110_-*b*-D_57_, (C) Q_110_-*b*-D_89_, and (D) Q_110_-*b*-D_219_. All samples were measured after 24 h equilibration time and N/P = 5.

DLS was employed to confirm the complexation between positively charged polymers and dsRNA and to assess the variability in the resulting size and polydispersity of the assembled polyplexes. DLS also confirmed that there is no sign of assembly of the diblock copolymers in aqueous solution, prior to interaction with the dsRNA (data not shown). Normalized intensity autocorrelation (IAC) data obtained for Q_110_, Q_110_-*b*-D_57_, Q_110_-*b*-D_89_, and Q_110_-*b*-D_219_ polyplexes with dsRNA at N/P ratio = 5 collected at three scattering angles are shown in Figure 3 (full IAC data shown in Figure S2 in the SI). They show the presence of a single relaxation mode and were indeed successfully fitted to eq 3 with *i* = 1
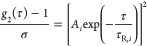
3where the coherence factor, σ, allows normalization of the data so that the *y*-intercept equals 1, and τ_R_ and *A_i_* are the relaxation time and the relative amplitude associated with the relaxation mode *i*, respectively.

**Figure 4:**
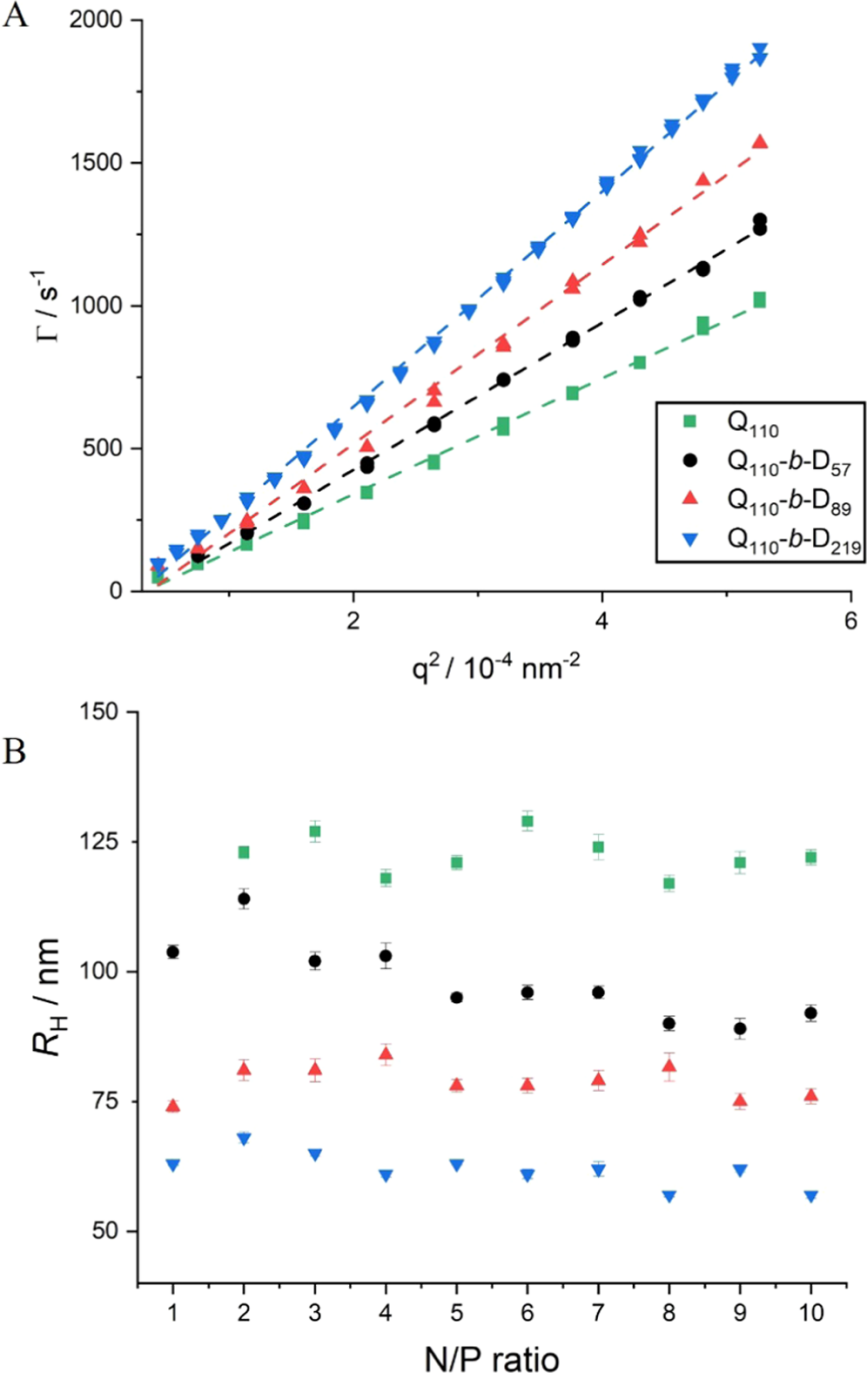
(A) Plots of the decay rate, Γ, as a function of the squared scattering vector, *q*^2^, for polyplexes of polymers with increasing PDMA chain lengths: Q_110_, Q_110_-*b*-D_57_, Q_110_-*b*-D_89_, and Q_110_-*b*-D_219_. Dashed lines are linear fits of the data and allow the determination of the diffusion coefficient *D* of the scattering objects (see eq 4). All samples were measured after 24 h equilibration time and N/P = 5. (B) Effective hydrodynamic radii, *R*_H_, of Q_110_/dsRNA, Q_110_-*b*-D_57_/dsRNA, Q_110_-*b*-D_89_/dsRNA, and Q_110_-*b*-D_219_/dsRNA polyplexes measured by dynamic light scattering, with N/P ratio = 1–10.

The obtained relaxation times, τ_R_, were then used to calculate the decay rates, , and subsequently plotted against the square of the scattering vector, *q*^2^, as shown in Figure 4A. Γ exhibits a *q*^2^-dependence, characteristic of a diffusive behavior. Hence, eq 4 was used to determine the diffusion coefficient with the non-null *y*-intercept, *B*, to account for the small uncertainties in the determination of Γ. Assuming a spherical shape for the measured objects, the Stokes–Einstein equation was subsequently used to calculate the hydrodynamic radius *R*_H_ (eq 5)
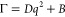
4
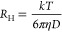
5The size of polyplex objects showed minimal variation over N/P ratio = 1–10 (Figure 4B in the SI; N/P ratios 1, 5, and 10 are highlighted in Figure S3 in the SI). Note that no *R*_H_ was determined at N/P ratio = 1 for Q_110_ polyplexes as large precipitated aggregates/clusters, visible to the naked eye, formed upon mixing at this particular N/P ratio. Instability of polyplexes made of two oppositely charged homopolymers around the isoelectric point is commonly reported in the literature,^50^ and our experiments further demonstrate the importance of incorporating a neutral block to sterically stabilize the polyplexes when close to charge neutrality. At higher N/P ratios, the polyplexes obtained with the homopolymer appeared to be stable, likely as a result of higher polyplex charge resulting from the imbalance of the overall number of charges between the polymer and the dsRNA, in agreement with previous literature.^51^

Figure 4 shows that as neutral block length is increased, the size of the polyplex objects decreases. The neutral PDMA block is incorporated to provide steric stabilization. It surrounds the electrostatically collapsed interpolyelectrolyte core comprising the dsRNA and PQDMAEMA, as previously predicted by coarse-grained simulation.^36^ Beyond steric stabilization, the hydrophilic corona formed by the PDMA block has the potential to provide further protection for the genetic material.^36^ It is important, however, to highlight that in all of these systems, the number of dsRNA chains within each of the polyplexes may not be constant, and this will play an important role in determining the size of the resulting polyplexes. Previous studies on the structure and morphology of interpolyelectrolyte complexes formed between cationic and anionic polyelectrolytes have revealed a dependence on the polymer composition with respect to neutral block length. Their findings show that incorporating a neutral block into one or both polyelectrolyte chains reduces aggregation and can shift complex structure from vesicles/worm-like cylinders toward star-shaped spherical morphologies.^52−54^ Petersen *et al.* studied bPEI-*g*-PEG/pDNA polyplexes and found that longer PEG blocks (with fewer grafted on) resulted in smaller sizes when complexed to pDNA.^40^ However, as far as the authors are aware, this is the first report of decreasing hydrodynamic radii with increasing neutral block length of DHBCs for polyplexes formed with dsRNA. The detailed morphology of the complexed DHBCs and dsRNA is not yet known, and future work will focus on high-resolution transmission electron microscopy and small-angle X-ray/neutron scattering to elucidate their in-depth structure.

### Electrophoretic Mobility of Polyplexes

**Figure 5:**
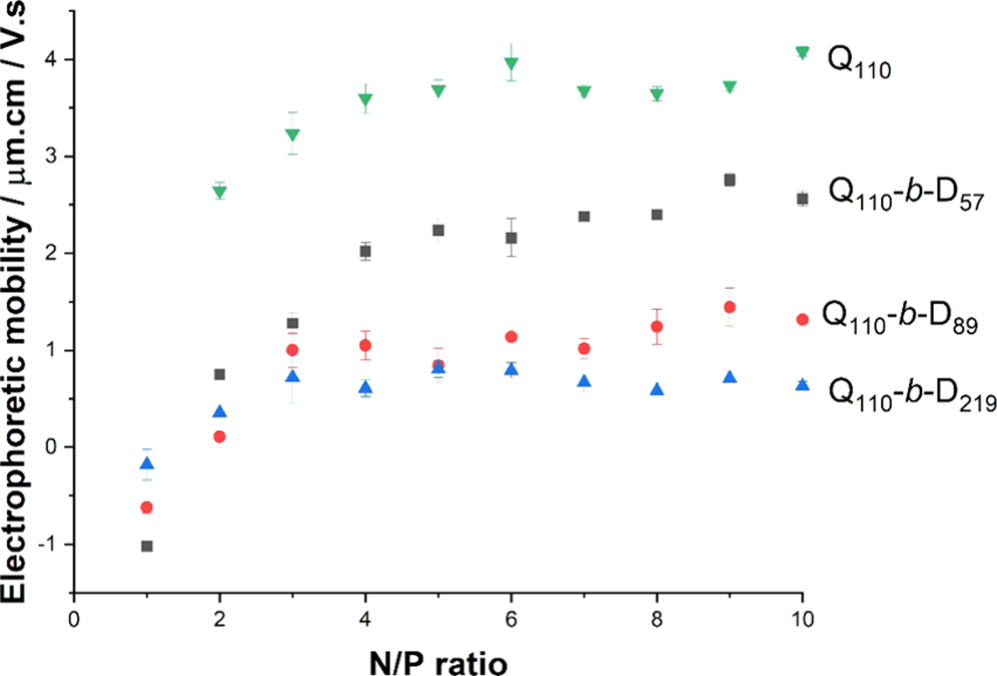
Electrophoretic mobility of polyplexes formed with each investigated polymer (Q_110_, Q_110_-*b*-D_57_, Q_110_-*b*-D_89_, and Q_110_-*b*-D_219_) over an N/P ratio range of 1–10. Note that no electrophoretic measurement was conducted on polyplexes formed with Q_110_ at N/P ratio = 1, where precipitation/aggregation was observed (see the previous discussion of DLS data).

The anionic backbone of dsRNA is a prohibitive factor for cell entry due to repulsive electrostatic interactions with negatively charged cell-surface glycosaminoglycans,^55^ whereas gene delivery vectors with a positive surface charge can promote genetic material entry into cells.^26,55−58^ However, cationic homopolymers can be cytotoxic if their surface charge is too high.^34^ Hence, a balance must be found to mitigate against toxicity by lowering cationic charge while still promoting the entry of dsRNA into cells. Thus, in this work, we measured the electrophoretic mobility (proportional to surface charge) of polyplexes as a function of the N/P ratio. As the N/P ratio was increased from 1 to 10, the polyplex electrophoretic mobility increased (Figure 5). At N/P ratio = 3, the electrophoretic mobility of Q_110_-*b*-D_89_/dsRNA and Q_110_-*b*-D_219_/dsRNA polyplexes is seen to plateau, likely as a result of no further polymer chain being added to the polyplexes. Samples with N/P ratios >3 are likely to contain free polycations. Q_110_/dsRNA and Q_110_-*b*-D_57_/dsRNA polyplexes reach this plateau at higher N/P ratios, suggesting that the polyplexes formed with these two polymers are, in comparison to Q_110_-*b*-D_89_/dsRNA and Q_110_-*b*-D_219_/dsRNA polyplexes, able to accommodate additional polymer chains in their structure at higher cationic polymer concentrations. In these cases, the additional chains incorporated within the Q_110_/dsRNA and Q_110_-*b*-D_57_/dsRNA polyplexes, relative to Q_110_-*b*-D_89_/dsRNA and Q_110_-*b*-D_219_/dsRNA polyplexes, are likely to increase polyplex particle size, which correlates with the larger *R*_H_ measured by DLS. At this stage, polyplexes made with Q_110_-*b*-D_89_ and Q_110_-*b*-D_219_ at N/P ratios ≥2 seem to be the best candidates for our application since electrophoretic mobility values are lower while still endowing a positive surface charge to aid endocytosis.

### Agarose Gel Retardation and EB Exclusion

The fluorophore, ethidium bromide (EB), intercalates between the base pairs of DNA/dsRNA. It weakly fluoresces in aqueous solution but exhibits a strong fluorescence when complexed with intact DNA or dsRNA.^59,60^

#### Agarose Gel Retardation

**Figure 6:**
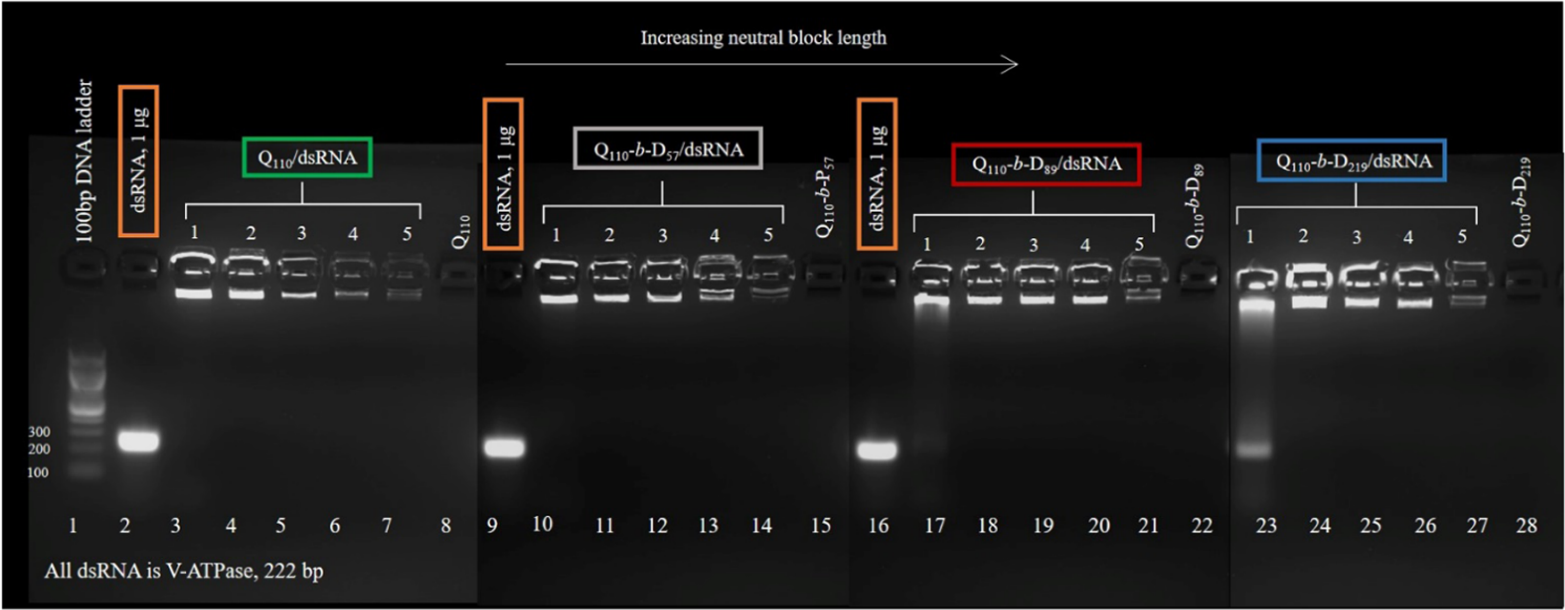
Agarose gel electrophoresis of polyplexes formed with each polymer (Q_110_ (lanes 3–7), Q_110_-*b*-D_57_ (lanes 10–14), Q_110_-*b*-D_89_ (lanes 17–21), and Q_110_-*b*-D_219_ (lanes 23–27)) over an N/P ratio range of 1–5. 100 bp DNA ladder was used in lane 1, dsRNA (1 μg) was added to lanes 2, 9, and 16, and polymers Q_110_, Q_110_-*b*-D_57_, Q_110_-*b*-D_89_, and Q_110_-*b*-D_219_ alone were added to lanes 8, 15, 22, and 28, respectively. These data were collected in four separate images of separate parts of the gel, so that a greater focus on the observed fluorescence could be obtained; hence, subtle changes in background colors between the images used can be seen.

Gel electrophoresis using agarose gel stained with EB was performed to assess the binding of polymers to dsRNA at N/P ratio = 1–5. Ordinarily, dsRNA inserted into the formed well at the top of the agarose gel will migrate to a specific location driven by the electric current. Successful complexation of dsRNA with a polymer can be confirmed by retardation of polyplexes, with fluorescence confined to the well of the corresponding gel lane, as opposed to the migration of free dsRNA. Where partial complexation occurs, a “smear” down the gel lane can be observed, as only a fraction of dsRNA chains are retarded against migration.^61^Figure 6 investigates the complexation of each of the polymers with increasing N/P ratios. For example, as indicated by the fluorescent wells, the homopolymer Q_110_ retards dsRNA migration at N/P ratios 1–5 (see Q_110_/dsRNA on the left side of Figure 6). The fluorescence, however, is also observed to decrease as the N/P ratio is increased. This is indicative of stronger binding to dsRNA and will be discussed in more detail in the next section.

Comparing Q_110_/dsRNA to polyplexes formed with the longest DHBC, Q_110_-*b*-D_219_, a difference can be seen at N/P ratio = 1. Q_110_-*b*-D_219_/dsRNA does not appear to retard the dsRNA migration as successfully as Q_110_/dsRNA, as illustrated by the smeared fluorescence down the gel lane. Overall, for DHBC-based polyplexes at N/P ratio = 1, as PDMA block length is increased, only partial complexation is achieved. A similar effect was identified by Lam *et al*. with PDMAEMA-*b*-poly(2-methacryloyloxyethyl phosphorylcholine) (MPC) diblock copolymers when complexed with plasmid DNA.^62^ As the length of the charge-neutral MPC block was increased, higher molar ratios of diblock copolymer were required to reach “full complexation”. Similarly, N/P ratios >1 are required for full complexation of all dsRNA chains by longer neutral block length polymers (Q_110_-*b*-D_89_ and Q_110_-*b*-D_219_). At N/P ratio = 1, the partial migration of dsRNA, indicated by a “smear” down the corresponding gel lane, was reproducible. Multiple gel electrophoresis runs specifically at N/P ratio = 1 were performed to confirm this (see Figure S4 in the SI).

#### EB Exclusion

**Figure 7:**
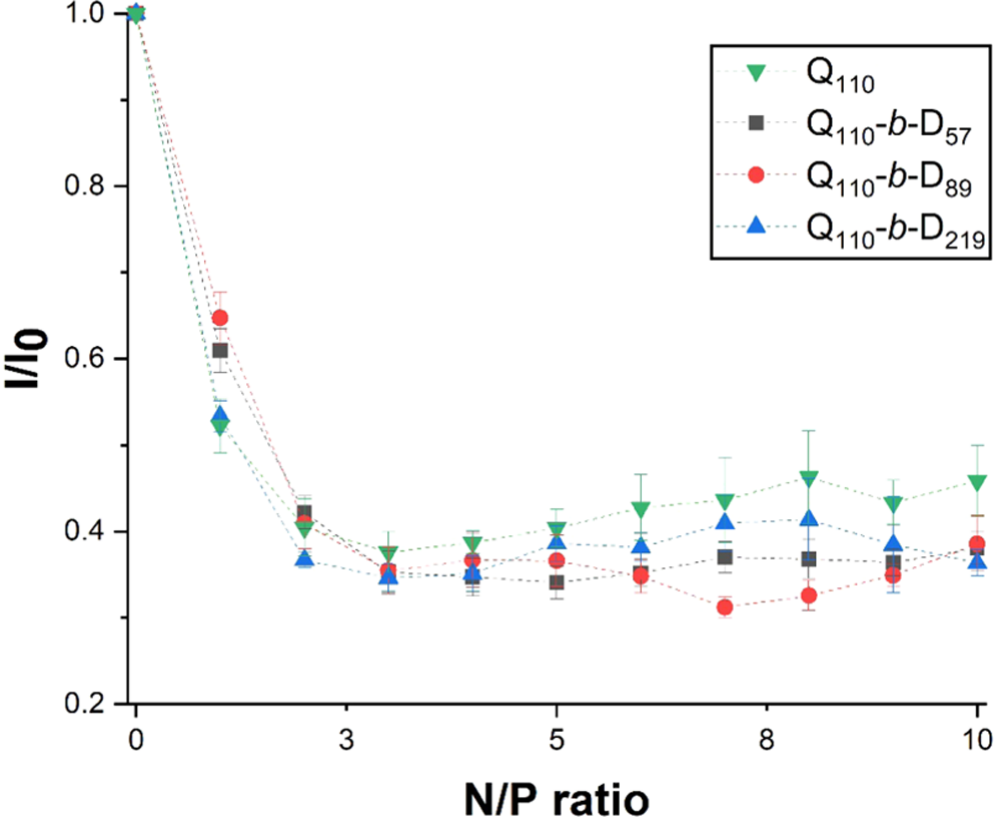
Ethidium bromide exclusion from dsRNA intercalation through the complexation of increasing amounts of polymers to dsRNA (increasing N/P ratio). Exclusion is quantitatively assessed through the quenching of fluorescence. Polyplexes formed with each polymer: Q_110_, Q_110_-*b*-D_57_, Q_110_-*b*-D_89_, and Q_110_-*b*-D_219_. Fluorescence intensity has been normalized with respect to the initial dsRNA–EB fluorescence. Lines are included to guide the eye only.

As qualitatively established in agarose gel electrophoresis, fluorescence of EB is quenched at higher N/P ratios. Through complexation of an interacting polycation with DNA/dsRNA, EB cannot intercalate as effectively, hence causing a decrease in fluorescence. As a result, it is possible to use quenching of fluorescence as a proxy for monitoring the polymer/dsRNA binding. This phenomenon has been well documented in the literature.^61,63−68^ Quenching of fluorescence can only be determined qualitatively using gel electrophoresis. Thus, fluorescence quenching titrations were performed *via* fluorescence spectroscopy over the N/P ratio = 0–10 (N/P ratio = 0 represents dsRNA–EB alone in aqueous solution and is used to quantify *I*_0_, fluorescence intensity at time = 0) to quantitatively interpret the exclusion of EB by each polymer. The proportion of quenched fluorescence is interpreted as an indicator of the strength of binding between the homopolymer/DHBC and the dsRNA. These data, presented in Figure 7, suggest that an N/P ratio = 3 is required for maximum fluorescence quenching. The error associated with the measurements indicates that there is no significant difference of the strength of binding between homopolymer and DHBCs with dsRNA. Complete EB exclusion (100% fluorescence quenching) is not achieved with Q_110_ nor DHBCs, which could imply that stronger binding may be possible with alternative polymer designs. For example, Dey *et al.* reported EB exclusion of 90–98% between ctDNA and either PMAPTAC homopolymer or a series of PMAPTAC-*b*-PEG diblock copolymers.^26^

### Influence of the Presence of Electrolyte on Polyplex Binding

**Figure 8:**
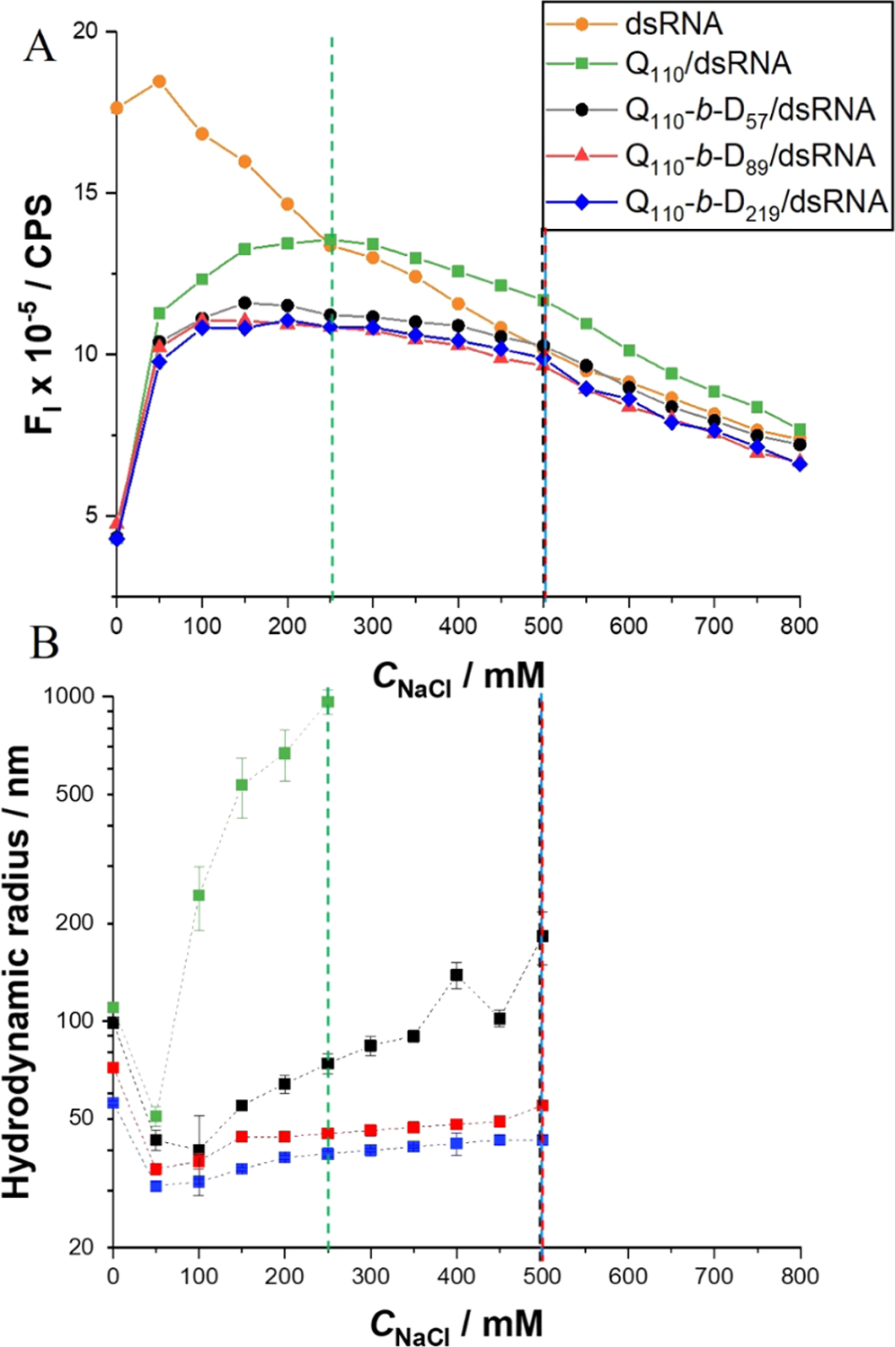
(A) Fluorescence intensity (a.u.) of polyplexes (N/P = 5), dsRNA, and EB with increasing *C*_NaCl_. DsRNA, in the absence of homopolymer or DHBC, is shown as the average spread of data following experiments run in quadruplicate. Lines are included to guide the eye only. (B) Average effective hydrodynamic radii (log scale) of Q_110_/dsRNA, Q_110_-*b*-D_57_/dsRNA, Q_110_-*b*-D_89_/dsRNA, and Q_110_-*b*-D_219_/dsRNA based on repeat measurements with increasing NaCl concentration. Dotted vertical lines are included to highlight crossover points.

When considering the final applications of the exogenous dsRNA material delivery in pharmaceutical/agrochemical formulations, one expects many inorganic ions to be present in these environments. To form a full understanding of these systems under these conditions, we have investigated the impact of NaCl concentration (*C*_NaCl_) on polyplex stability. As demonstrated by fluorimetric titration (Figure S5 in the SI and Figure 8), increasing concentration of NaCl in the presence of dsRNA–EB induces a decrease in the fluorescence intensity, as previously shown in the literature.^63^

Binding of EB to nucleic acids occurs primarily through the intercalation of base pairs. However, there is also a contribution to binding *via* electrostatic interaction of the cationic amine site of EB with the anionic phosphate groups. The addition of an electrolyte weakens the binding of EB through electrostatic interaction, thus leading to a decrease in the fluorescence intensity of dsRNA–EB with increasing electrolyte concentration. It is worth noting that this phenomenon has been shown to have a larger impact on dsRNA than on DNA.^59,60,65,69^ The fluorescence intensity of polyplexes formed with dsRNA is plotted as a function of the NaCl concentration in Figure 8A. To explain the behavior of these systems, we analyze the results alongside size analysis (see Figure S6 in the SI) to determine the decomplexation point. Prior to the addition of NaCl to polyplex formulations, the relative fluorescence intensity of the polyplexes is low as a result of the displacement of EB from dsRNA.^61,63,65−67,69^

The increase in fluorescence intensity in the region where *C*_NaCl_ = 50–200 mM is a result of increased binding of EB to dsRNA. Transitioning from a salt-free environment to *C*_NaCl_ = 50 mM, polyplexes appear to form smaller units due to chain rearrangement with the decrease in Debye length. Similar effects in polyelectrolyte complexes have been observed in the literature.^70−72^ The rearrangement allows increased EB access to intercalate with dsRNA, hence the large increase in fluorescence intensity.

From *C*_NaCl_ = 50 mM, light scattering reveals an increase in polyplex size due to the electrostatic screening of charge by the addition of competing cations and anions. Charge screening causes swelling of the polyplexes due to osmotic repulsion.^73^ This size increase is more pronounced for polyplexes formed by polymers with shorter or no PDMA neutral blocks. Petersen *et al.* also found that polyplexes formed between bPEI-*g*-PEG and pDNA swelled in size at *C*_NaCl_ = 150 mM.^40^

Full decomplexation can be characterized as the crossover point beyond which the polyplex samples mimic dsRNA–EB fluorescence, with decreasing intensity upon further increase in *C*_NaCl_.^63^ Light scattering confirms the instability of polyplexes at this crossover point, beyond which multimodal size distributions are observed (data not shown) and DLS data can no longer be exploited. Q_110_/dsRNA reaches the crossover point at *C*_NaCl_ ∼ 250 mM, whereas DHBC/dsRNA does not reach that point until *C*_NaCl_ ∼ 500 mM.

The swelling regime of Q_110_/dsRNA (*C*_NaCl_ = 50–250 mM) has greater fluorescence intensity than the equivalent regime (*C*_NaCl_ = 100–500 mM) in DHBC/dsRNA samples. This implies an increased proportion of dsRNA available for EB intercalation. The addition of the steric-stabilizing PDMA block may therefore play a role in maintaining a greater degree of binding with dsRNA as *C*_NaCl_ is increased, particularly as full decomplexation of DHBC/dsRNA is not achieved until *C*_NaCl_ = 500 mM.

The mammalian intracellular Na^+^ and Cl^–^ concentrations are 10–12 and 4 mM, respectively, with extracellular concentrations of 145 and 116 mM, respectively.^74,75^ According to fluorimetric titration and light scattering measurements, full decomplexation of polyplexes does not occur until *C*_NaCl_ ∼ 250–500 mM. Therefore, our data suggest that there is potential for these formulations to provide adequate protection of dsRNA for *in vivo* applications.

### Protection of dsRNA against Enzymatic Degradation

A major barrier to the successful delivery of exogenous genetic material in therapeutic or agrochemical applications is overcoming the fragility of dsRNA to environmental nucleases. RNase A cleaves dsRNA after every cytosine and uracil and was thus used to investigate the enzymatic degradation and/or protection of dsRNA.

**Figure 9:**
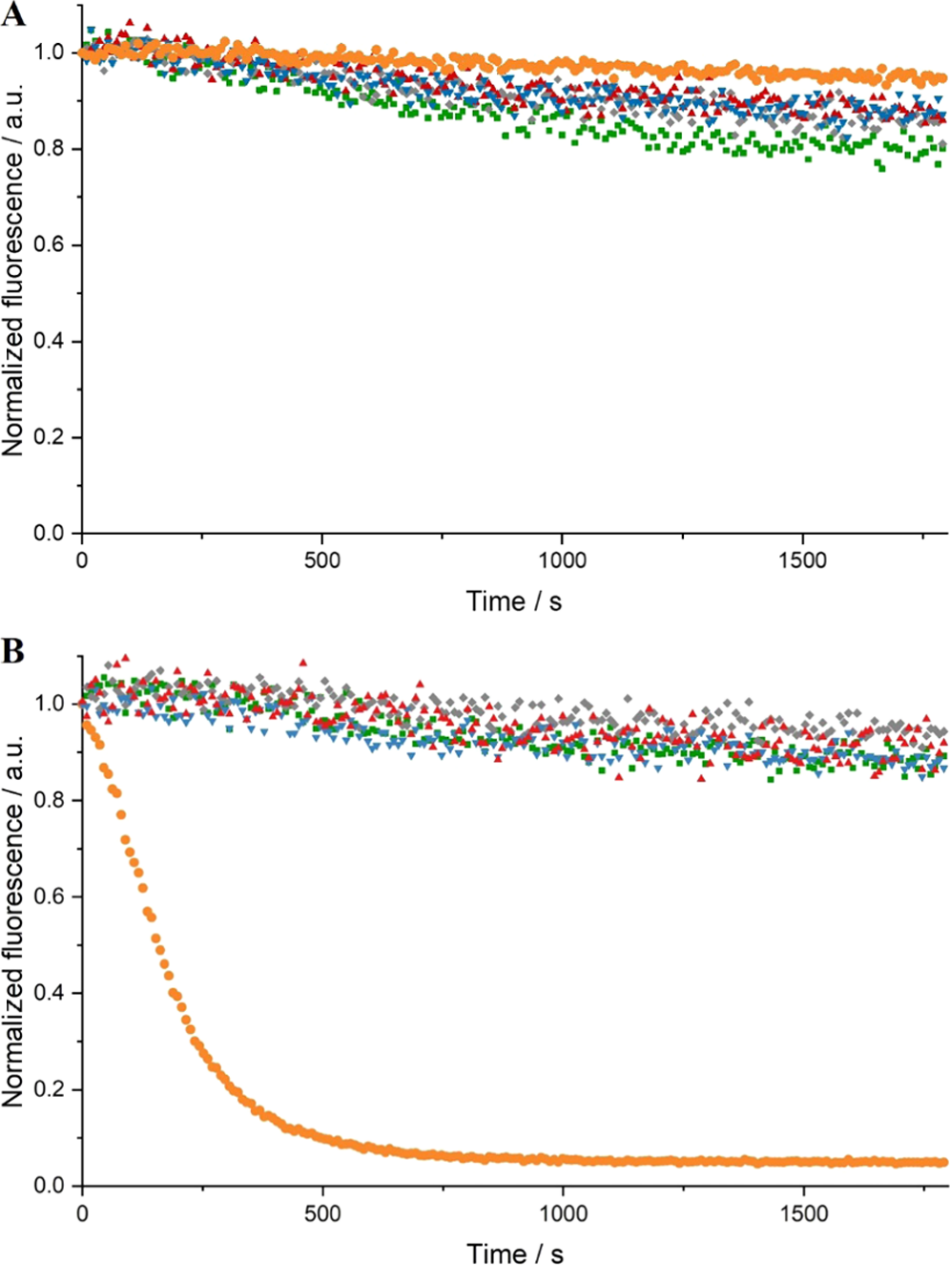
Time-resolved normalized fluorescence spectroscopy of dsRNA (orange), Q_110_/dsRNA polyplex (green), Q_110_-*b*-D_57_/dsRNA polyplex (gray), Q_110_-*b*-D_89_/dsRNA polyplex (red), and Q_110_-*b*-D_219_/dsRNA polyplex (blue), without (A) and with (B) the addition of RNase A. Polyplexes were left for 1.5 h to equilibrate. Data normalized with respect to *I*_0_ (fluorescence intensity at time = 0). Non-normalized data in the SI (Figure S7) show the low fluorescence intensity of polyplexes due to quenching of EB fluorescence after displacement from dsRNA intercalation.

Time-resolved fluorescence spectroscopy quantitatively highlights the dramatic, rapid degradation of dsRNA by RNase A (Figure 9; see Figure S7 in the SI for raw data). Polyplex fluorescence upon the addition of RNase A shows minimal difference to polyplex samples without RNase A. To further investigate the level of protection of dsRNA in polyplexes, complexation and proportion of degradation were qualitatively assessed in agarose gel electrophoresis assays (Figures S8–S11 in the SI). The outcomes of these experiments are summarized in Table 2, which indicates the complexation state (none, partial, or full) of dsRNA with homopolymer or DHBCs at specified N/P ratios, as well as whether dsRNA was degraded through the addition of RNase A. Where partial degradation is described, it corresponds to the degradation of dsRNA that was not complexed in the control samples. Overall, Q_110_ and DHBCs provide full protection to dsRNA against degradation by RNase A at N/P ratios ≥2, which is promising for therapeutic and agrochemical applications.

**Table 2:** Summary of Complexation and Protection Provided to dsRNA by Polymers at Different N/P Ratios, as Evaluated by Agarose Gel Electrophoresis (Figures S8–S11 in the SI)

N/P ratio		0	1	≥2
dsRNA	dsRNA degraded in the presence of RNase A	yes	n/a	n/a
Q_110_/dsRNA	Complexation level	n/a	partial	full
	dsRNA degraded in the presence of RNase A	n/a	yes	no
Q_110_-*b*-D_57_/dsRNA	Complexation level	n/a	partial	full
	dsRNA degraded in the presence of RNase A	n/a	yes	no
Q_110_-*b*-D_89_/dsRNA	Complexation level	n/a	partial	full
	dsRNA degraded in the presence of RNase A	n/a	yes	no
Q_110_-*b*-D_219_/dsRNA	Complexation level	n/a	partial	full
	dsRNA degraded in the presence of RNase A	n/a	yes	no

## Conclusions

In this work, we have successfully synthesized a series of double hydrophilic block copolymers *via* RAFT polymerization in aqueous media containing a cationic PQDMAEMA block and a neutral PDMA block of varying lengths. The electrostatic interaction between DHBCs, or cationic homopolymer, with 222 bp V-ATPase dsRNA induces the formation of polyplexes that retard the migration of the nucleic acid through agarose gel. Increasing the length of the charge-neutral PDMA block was identified to have an inverse relation to the hydrodynamic radii (*R*_H_) of polyplexes when characterized by DLS. The absence of a neutral block led to the largest-size (*R*_H_ ∼ 120 nm) polyplexes, whereas the longest PDMA block DHBC formed the smallest-size (R_H_ ∼ 60 nm) polyplexes with dsRNA. As N/P ratio was varied, there was no significant impact on polyplex size. However, when formulating at a 1-to-1 charge ratio (N/P ratio = 1), a neutral PDMA block is required to sterically stabilize polyplexes to prevent aggregation and precipitation. The results reported here suggest that longer PDMA block DHBCs require higher N/P ratios (increased amount of polymer) to fully complex all dsRNA, with partial complexation at N/P ratio = 1 qualitatively identified in agarose gel electrophoresis assays. DLS data and electrophoretic mobility assays indicate that when PDMA length is increased, more compact polyplexes are formed with dsRNA. The cationic homopolymer and all DHBCs successfully protected dsRNA against degradation by RNase A when complexed at N/P ratio ≥ 2. We thus believe that these formulations show promising potential as nonviral delivery vehicles for dsRNA. Incorporating a neutral steric-stabilizing polymer block protects against full decomplexation in the presence of competitive salt ions until *C*_NaCl_ = 500 mM. Therefore, the designed double hydrophilic block copolymers present interesting candidates for dsRNA delivery applications.

## Supporting Information Available

Polymer characterization by ^1^H NMR spectroscopy, complete DLS data from all polyplexes formulated at N/P ratio = 5, effective hydrodynamic radii at N/P ratios 1, 5, and 10 from DLS, agarose gel electrophoresis repeats of N/P ratio = 1 polyplex samples, fluorimetric titration of dsRNA–EB with increasing *C*_NaCl_, DLS IAC curves from the *C*_NaCl_ titration assay, raw fluorescence intensity data from RNase A degradation assays, and complexation/protection assays by agarose gel electrophoresis (PDF)

## Abbreviations

RNAi: RNA interference
dsRNA: double-stranded RNA
PQDMAEMA: poly(2-dimethylaminoethyl methacrylate)
PDMA: poly(*N,N*-dimethylacrylamide)
CCCP: 4-((((2-carboxyethyl)thio)carbonothioyl)thio)-4-cyano-pentanoic acid
ACVA: 4,4′-azobis(4-cyanovaleric acid)
DLS: dynamic light scattering
GPC: gel permeation chromatography
DHBC: double hydrophilic block copolymer
EB: ethidium bromide
