# Supplementary material for: Protection of Double-Stranded RNA via Complexation with Double Hydrophilic Block Copolymers: Influence of Neutral Block Length in Biologically Relevant Environments
Source: Biomacromolecules. 2022 May 12;23(6):2362–73. doi: 10.1021/acs.biomac.2c00136 (PMC9198985; doi:10.1021/acs.biomac.2c00136)
Supplement: Supplementary file 1 — bm2c00136_si_001.pdf [file bm2c00136_si_001.pdf]

Supplementary Information to

Protection of Double Stranded-RNA *via* Complexation with Double Hydrophilic Block Copolymers: Influence of Neutral Block Length in Biologically Relevant Environments

Charlotte E. Pugsley[1,2], R. Elwyn Isaac[2], Nicholas. J. Warren[1], Juliette S. Behra[1,†] , Kaat Cappelle[3], Rosa Dominguez-Espinosa[4], and Olivier. J. Cayre[1,*]*

[1]School of Chemical and Process Engineering, University of Leeds, Leeds, LS2 9JT, United Kingdom

[2]School of Biology, Faculty of Biological Sciences, University of Leeds, Leeds, LS2 9JT, United Kingdom

[3]Ghent Innovation Center, Technologiepark 30, B-9052 Gent-Zwijnaarde, Belgium

[4]Syngenta Jealott's Hill International Research Centre, Bracknell, Berkshire, RG42 6EY, United Kingdom

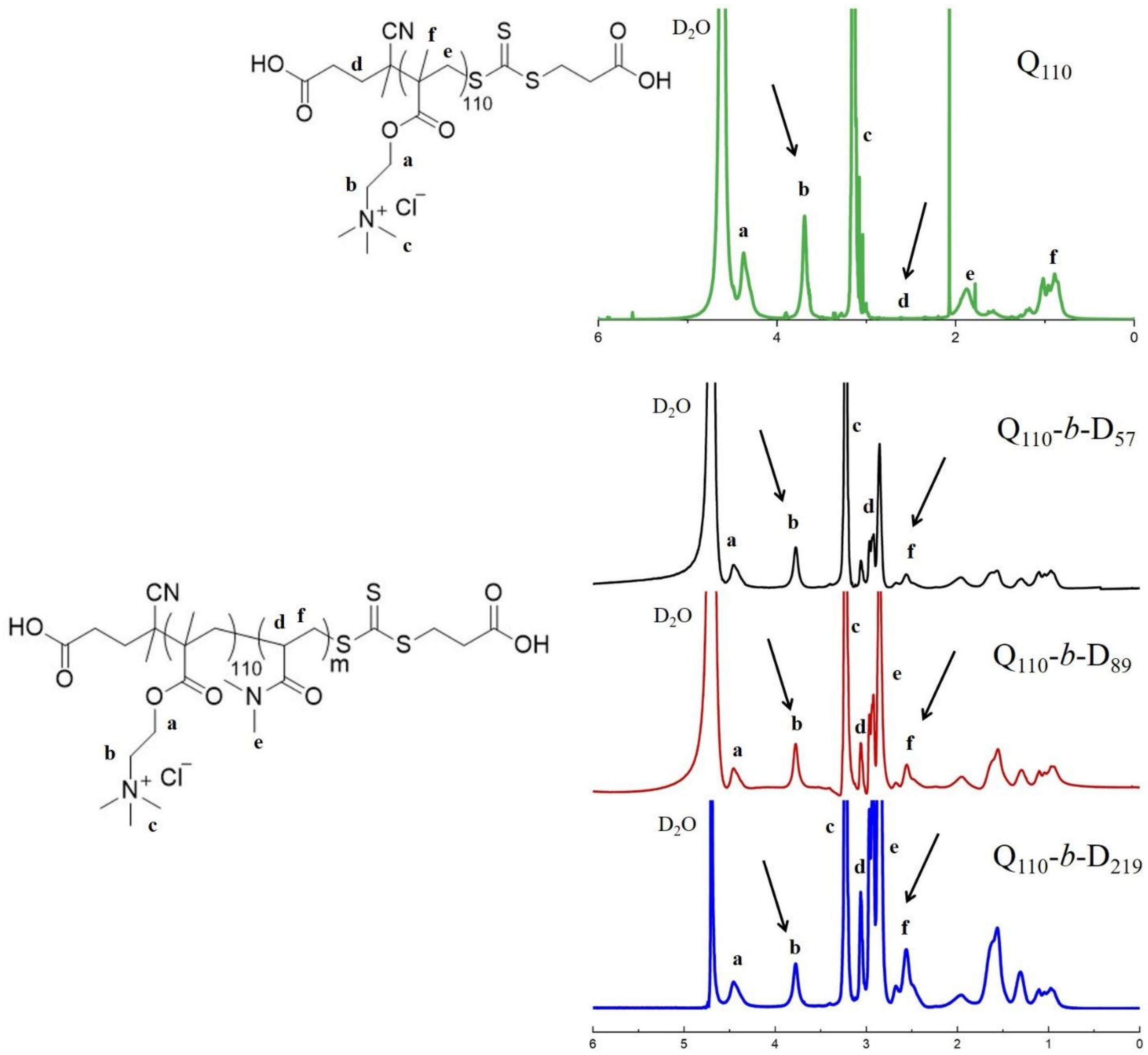

Figure S1. ^{1}H NMR spectra (400 MHz) of homopolymer Q_{110} (upper part) and diblock copolymers Q_{110}-b-D_{57}, Q_{110}-b-D_{89} and Q_{110}-b-D_{219} (lower part) following purification by dialysis, subsequent lyophilization, and dissolution in D_2O at polymer concentrations of 5 mg mL^{-1}. Comparison of the backbone peak **d** with peak **b** was used to confirm the degree of polymerization of the PQDMAEMA macro-chain transfer agent. Subsequently, the relationship between areas of peak **b** and peak **f** was used to determine the degree of polymerization of PDMA in diblock copolymers.

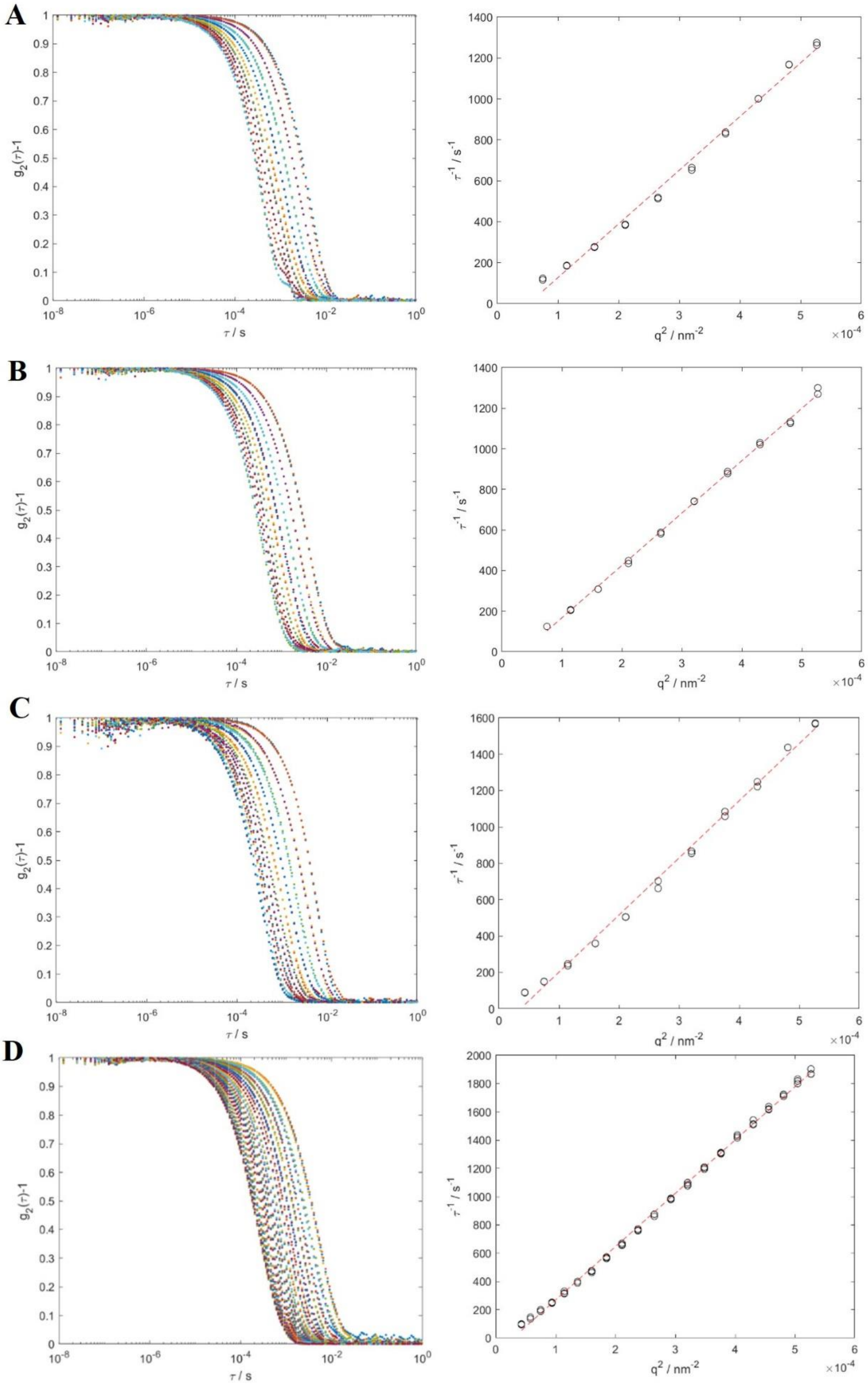

Figure S2. DLS data collected on samples formulated at N/P ratio = 5 after 24 h equilibration time. (Left) Examples of normalized intensity auto-correlation (IAC) data measured by DLS at scattering angles 30 – 130°. Angle increases from right to left, with measurements in triplicate (not all visible due to overlap of similar data points). (Right) Plots of the decay rate, Γ, against the squared scattering vector, q^2. Dashed lines are fits to $\Gamma = Dq^2 + B$, where the non-null y-intercept, B, to account for a small uncertainty in the values of Γ. (A) Q_{110}/dsRNA, (B) Q_{110}-*b*-D_{57}/dsRNA, (C) Q_{110}-*b*-D_{89}/dsRNA, (D) Q_{110}-*b*-D_{219}/dsRNA.

Polyplex	Effective hydrodynamic radii, R_H / nm		
	N/P ratio		
	1	5	10
Q_{110}	*	121	122
Q_{110}-*b*-D_{57}	104	95	92
Q_{110}-*b*-D_{89}	74	78	76
Q_{110}-*b*-D_{219}	63	63	57

Table S3. Effective R_H of polyplexes formed by Q_{110} and DHBCs with dsRNA at N/P ratios of 1, 5 and 10. * = visible aggregation occurred, LS measurement not possible.

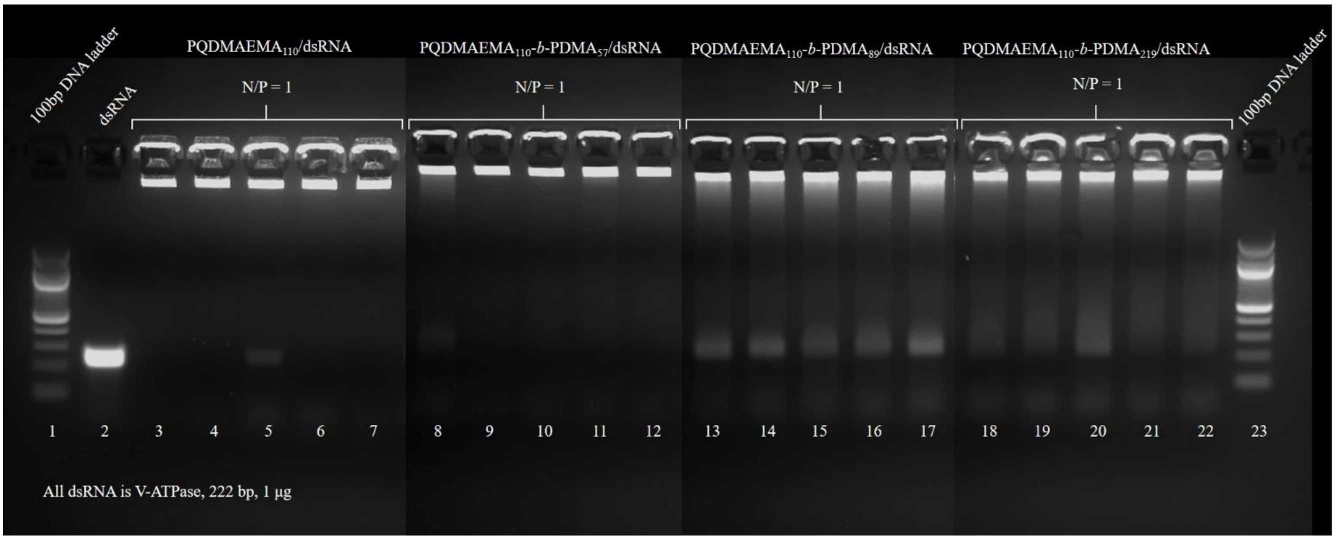

Figure S4. Agarose gel electrophoresis replicates of N/P ratio = 1 samples of Q_{110}/dsRNA, Q_{110}-*b*-D_{57}/dsRNA, Q_{110}-*b*-D_{89}/dsRNA and Q_{110}-*b*-D_{219}/dsRNA polyplexes. These data were collected in four separate images of separate parts of the gel, so that a greater focus on the observed fluorescence could be obtained; hence, subtle changes in background colors between the images used can be seen.

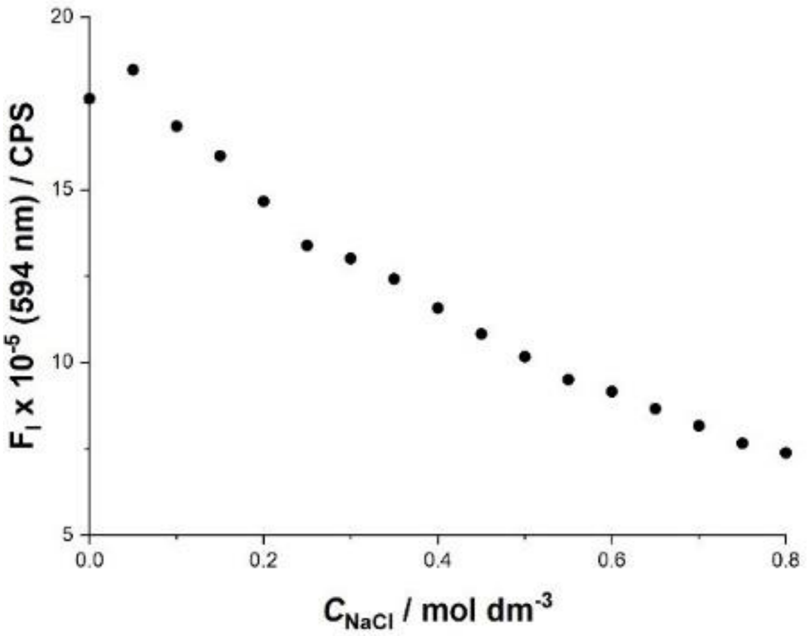

Figure S5. Fluorimetric titration of dsRNA-EB with increasing C_{NaCl} in a homopolymer and DHBC-free environment.

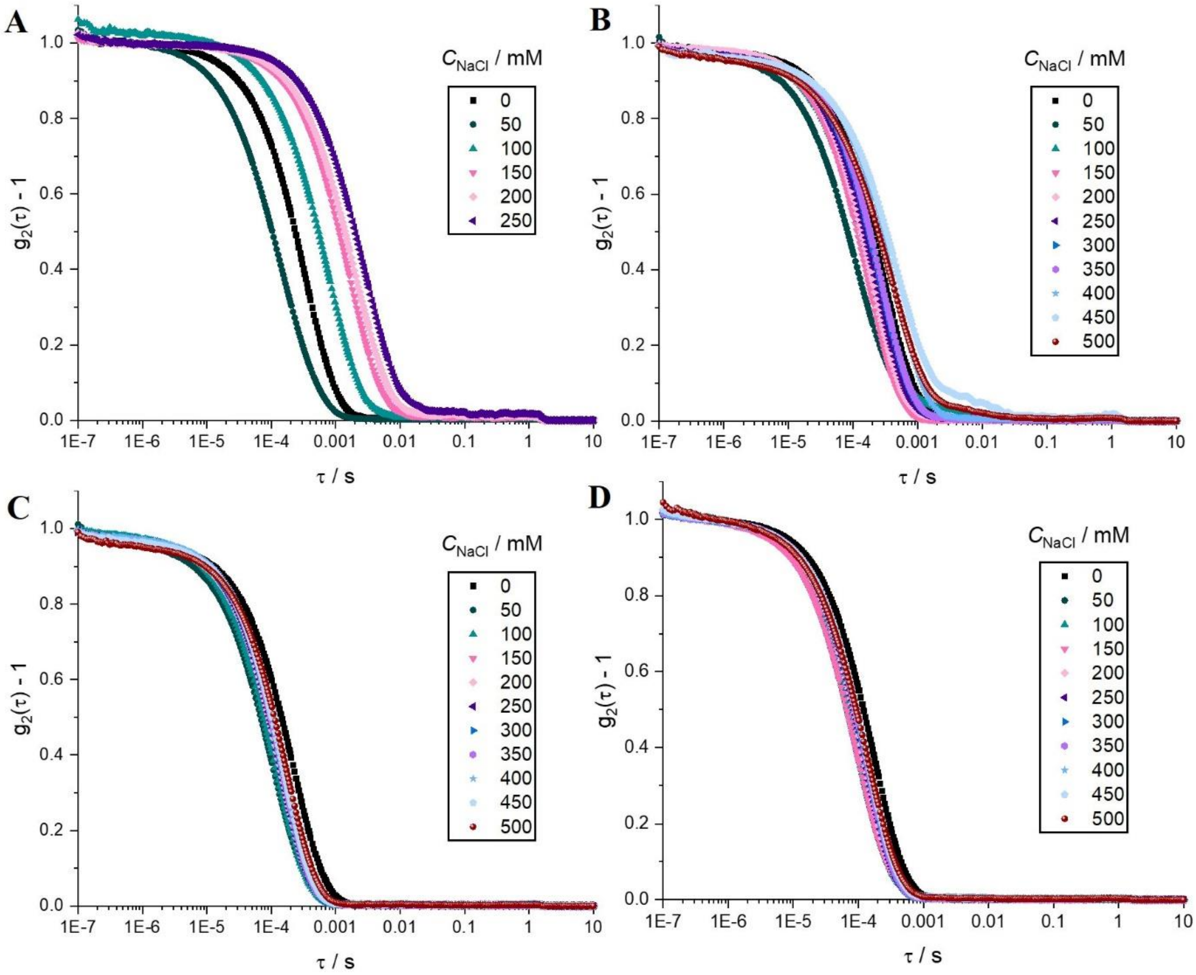

Figure S6. Dynamic light scattering intensity auto-correlation data from NaCl titration assay collected with zeta potential analyzer showing an increase in relaxation time with increasing NaCl concentration. Fits performed using the following equation:

$$\frac{g_2(\tau)-1}{\sigma} = \left[\sum_i A_i exp\left(-\frac{\tau}{\tau_{R,i}}\right)\right]^2$$

Where the coherence factor, σ, allows normalization of the data so that the y-intercept equals 1, $\tau_{R,i}$ is the relaxation time of the relaxation mode i and A_i is its associated relative amplitude. Fits using a single exponential function (i = 1) were used in most cases, except when a second relaxation mode of small amplitude was observed (*e.g.* data collected in 250 mM NaCl shown in (A), where a sum of two exponential functions (i = 2) was used). (A) Q_{110}/dsRNA, (B) Q_{110}-*b*-D_{57}/dsRNA, (C) Q_{110}-*b*-D_{89}/dsRNA and (D) Q_{110}-*b*-D_{219}/dsRNA.

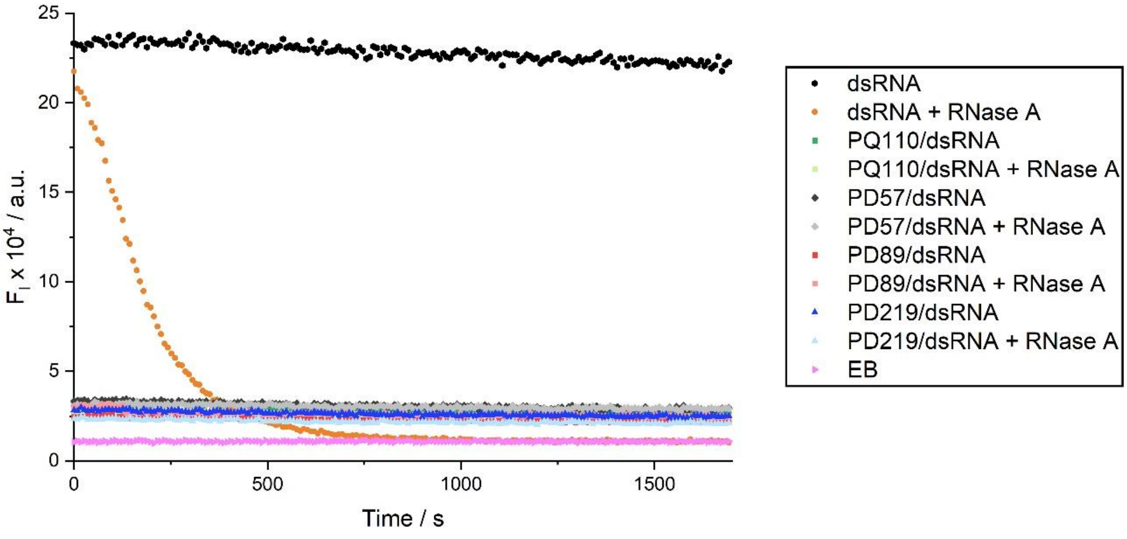

Figure S7. Fluorescence intensity raw data from RNase A degradation assay. Due to EB displacement, polyplex fluorescence remains significantly quenched throughout the assay, hence values are normalized to I_0.

In the following agarose gel electrophoresis assays (Figures S7–S10) 1 µg dsRNA was added per lane. Complexation and proportion of degradation were qualitatively assessed in agarose gel electrophoresis assay and are reported in Table 2 of the main manuscript. Partial complexation was inferred by a smeared gel lane in the absence of RNase A. Partial degradation occurred when complexation was not full (*i.e.* smeared gel lane in absence of RNase A) hence un-complexed dsRNA was degraded by RNase A and the smear down the gel lane is no longer visible, but some fluorescence remains in the well.

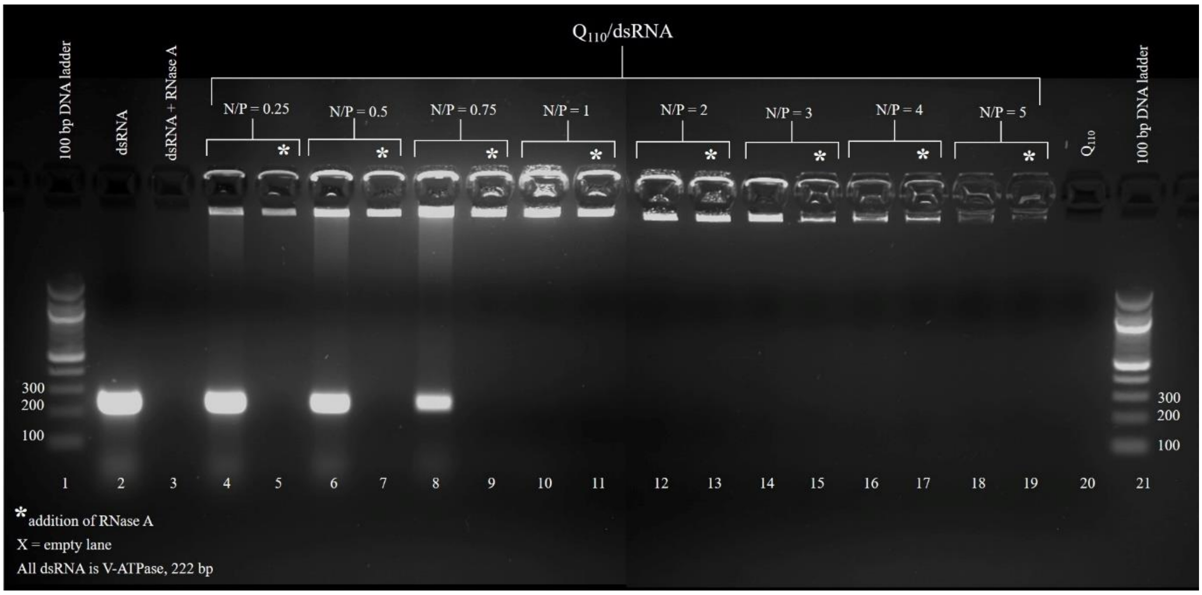

Figure S8. Agarose gel electrophoresis of Q_{110}/dsRNA polyplexes at N/P ratio = 0.25, 0.5, 0.75 1, 2, 3, 4 and 5, with and without the presence of RNase A enzyme. These data were collected in two separate images of separate parts of the gel, so that a greater focus on the observed fluorescence could be obtained; hence, subtle changes in background colors between the images used can be seen.

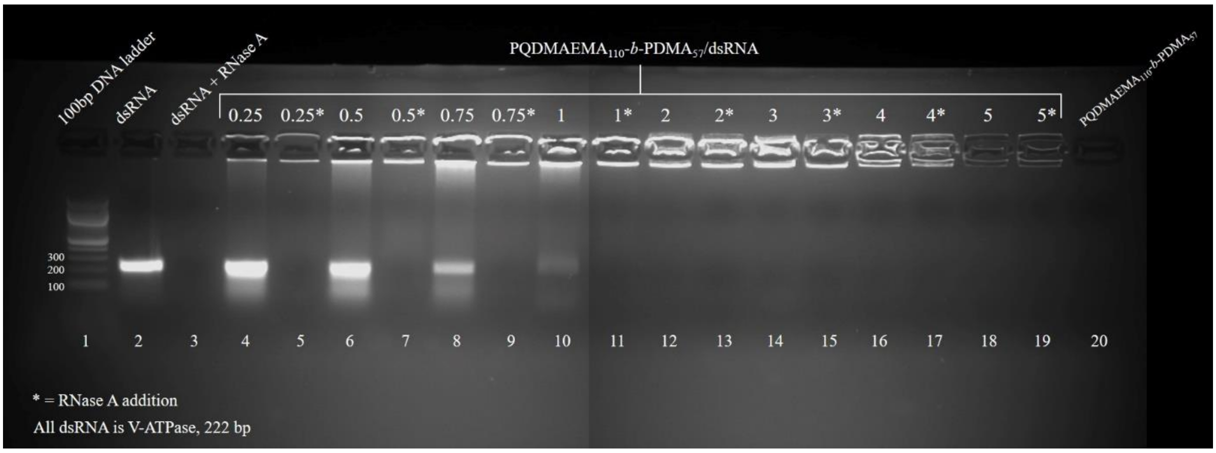

Figure S9. Agarose gel electrophoresis of Q_{110}-*b*-D_{57}/dsRNA polyplexes at N/P ratio = 0.25, 0.5, 0.75, 1, 2, 3, 4 and 5, with and without the presence of RNase A enzyme. These data were collected in two separate images of separate parts of the gel, so that a greater focus on the observed fluorescence could be obtained; hence, subtle changes in background colors between the images used can be seen.

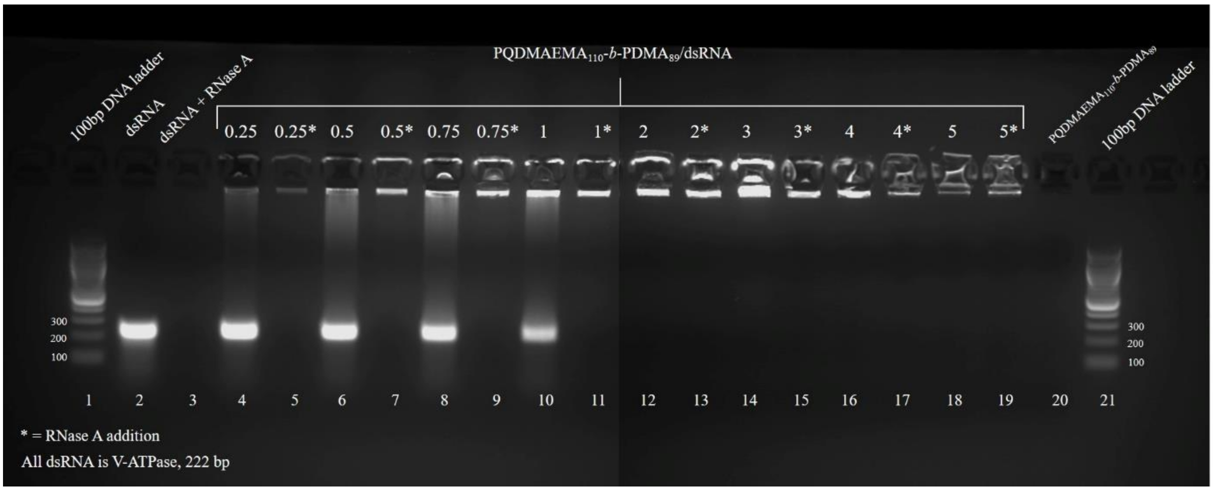

Figure S10. Agarose gel electrophoresis of Q_{110}-*b*-D_{89}/dsRNA polyplexes at N/P ratio = 0.25, 0.5, 0.75, 1, 2, 3, 4 and 5, with and without the presence of RNase A enzyme. These data were collected in two separate images of separate parts of the gel, so that a greater focus on the observed fluorescence could be obtained; hence, subtle changes in background colors between the images used can be seen.

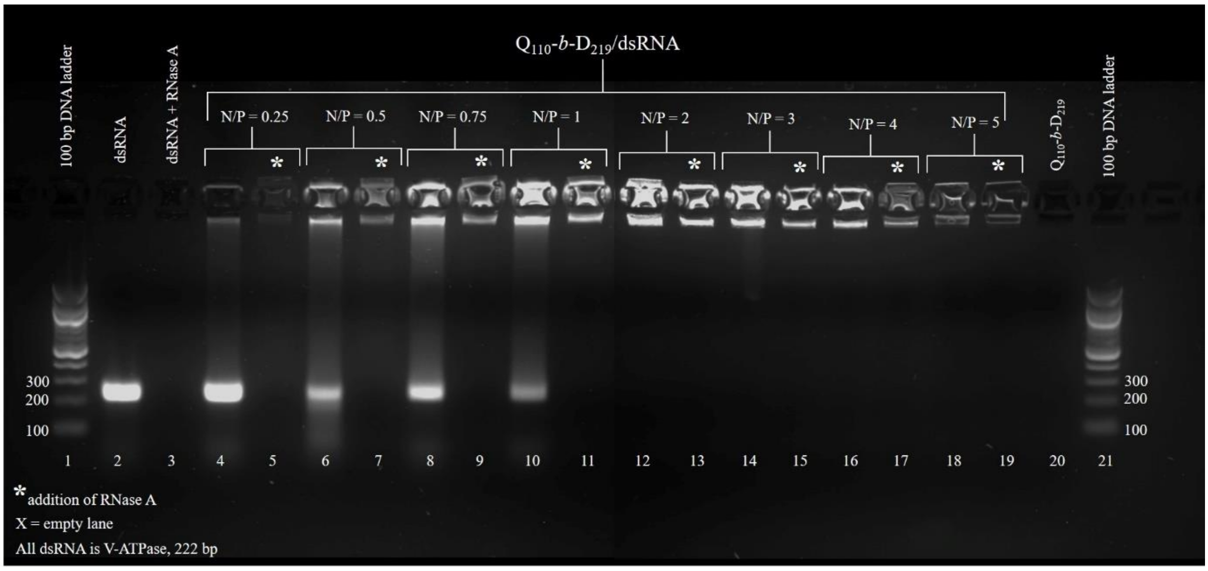

Figure S11. Agarose gel electrophoresis of Q_{110}-*b*-D_{219}/dsRNA polyplexes at N/P ratio = 0.25, 0.5, 0.75, 1, 2, 3, 4 and 5, with and without the presence of RNase A enzyme. These data were collected in two separate images of separate parts of the gel, so that a greater focus on the observed fluorescence could be obtained; hence, subtle changes in background colors between the images used can be seen.

Present Addresses

†Institute of Material Science, Nestle Research, CH-1000, Lausanne 26, Switzerland
